# Epidemiology, clinical manifestation, diagnosis,and treatment of bursitis iliopectinea: A systematic review

**DOI:** 10.1177/20503121251317899

**Published:** 2025-02-03

**Authors:** Malgorzata Lea Jonczy, Lorenz Büchler, Yadusha Mahenthiran, Fabrice Helfenstein, Christian Appenzeller-Herzog, Andrej Isaak

**Affiliations:** 1 Faculty of Medicine, University of Basel, Basel, Switzerland; 2 Department of Orthopaedic Surgery and Traumatology, Kantonsspital Aarau, University of Bern, Aarau, Switzerland; 3 Department of Orthopedic Surgery and Traumatology, Inselspital, Bern University Hospital, University of Bern, Switzerland; 4 Department of Internal Medicine, Spital Langenthal, Langenthal, Switzerland; 5 Deparment of Clinical Research, University of Bern, Switzerland; 6 Division of Angiology, Department of Clinical Research, University of Bern, Swiss Cardiovascular Center, Inselspital, Bern University Hospital, Bern, Switzerland; 7 University Medical Library, University of Basel, Basel, Switzerland; 8 Department of Vascular Surgery, University Hospital Basel, Basel, Switzerland

**Keywords:** Iliopsoas bursitis, hip arthritis, neurovascular compression, ultrasound diagnosis, total hip arthroplasty

## Abstract

**Background:**

Bursitis iliopectinea (BI) is a condition that is characterized by swelling of the iliopsoas bursa, leading to compression of inguinal neurovascular structures, causing swelling, pain, paresthesia, or thrombosis of the leg.

**Questions:**

Due to the rare occurrence of BI, the available literature consists of case reports. Our study aims to systematically review the literature for a comprehensive analysis of the etiology, treatment modalities, and clinical outcomes of patients with BI.

**Methods:**

We systematically analyzed 217 studies with 502 cases of BI and extracted information about the terminology, risk factors, diagnostic and treatment strategies, association with neurovascular compression syndromes, treatment outcomes, and recurrence rates.

**Results:**

The overall quality of the analyzed studies was moderate to good. The terminology uses for BI was heterogeneous and included ganglion, tumor, mass, and bursitis. In addition to conventional X-ray, ultrasound, CT, or MRI were used to diagnose BI. The most prevalent etiology of BI was osteoarthritis of the hip or wear-related soft-tissue reactions after total hip replacement (THA). Nearly one-third of the patients suffered from compression syndromes, most frequently of the femoral vein (16%). Only rheumatoid arthritis showed an association with the occurrence of compression syndromes. The most common operative treatments were the resection of the bursa (30%), total hip arthroplasty (29%), and aspiration (24%). Use of analgesics (17%), injection of corticoids (11%), and physiotherapy (9%) were used for conservative treatments. The recurrence rate was highest after physiotherapy (OR: 4.1) or aspiration (4.5) and lowest after THA (OR: 0.2).

**Conclusions:**

Although BI is a condition commonly associated with hip arthritis or local tissue reactions following total hip prosthesis, its impact extends beyond typical hip-related symptoms. Notably, BI related to rheumatoid arthritis shows a high correlation with neurovascular compression symptoms, with femoral vein compression being the most frequently reported. This underscores the necessity of considering BI in patients presenting with nonspecific inguinal pain or neurovascular symptoms of the lower extremity. Additionally, standardizing the nomenclature of BI nomenclature could improve future research.

## Introduction

The iliopsoas bursa is the largest synovial bursa in the body,^1–3^ present bilaterally in 98% of the adult population, often communicating with the hip joint. It extends from the iliopubic eminence to the anterior hip joint to reduce friction between the iliopsoas tendon and the ilium.^1,2^

Bursitis iliopectinea (BI) is a rare and under-recognized condition that can cause hip pain and inguinal swelling.^4^ Two primary mechanisms have been postulated in its development: increased synovial fluid pressure in the hip joint due to arthritis^2^ or inflammation caused by mechanical irritation, foreign bodies,^2^ acute trauma, or overuse.^5^ Associated conditions include crystal arthropathies, pigmented villonodular synovitis, synovial chondromatosis^5^ as well as polyethylene or metal-on-metal debris after hip arthroplasty.^3,6,7^ Among patients with symptomatic hip osteoarthritis, the prevalence of BI is estimated between 2% and 6.8%.^1,8^

The nonspecific symptoms and rarity of BI can lead to delayed or missed diagnosis.^5^ Compression of the inguinal neurovascular structures by an enlarged iliopsoas bursa can cause significant complications, including groin pain, neuropathic symptoms, limb swelling, deep vein thrombosis, or lower extremity ischemia.^5,9^ Accurate and timely diagnosis is essential for guiding effective treatment strategies.

**Figure 1.**
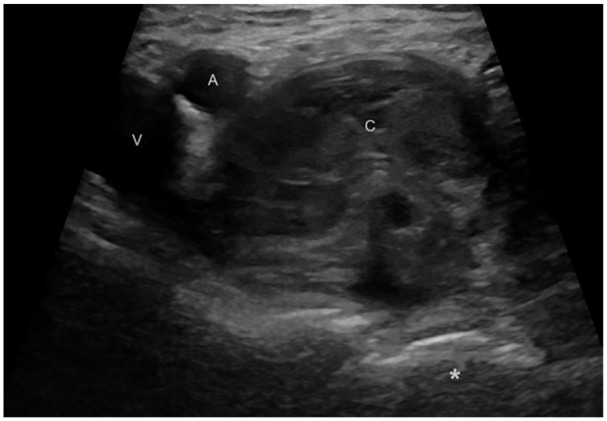
Ultrasound image, cross-sectional view at the level of the inguinal ligament. The femoral artery (a) and common femoral vein (v) are identified, along with a cystic mass (c) adjacent to these structures. The femoral head is marked with an asterisk (*).

Imaging plays a crucial role in diagnosing BI. Ultrasonography (US) is often the first-line imaging modality because of its accessibility, cost-effectiveness, and ability to quickly visualize the iliopsoas bursa (see also Figure 1). High-resolution ultrasound (HRUS) using high-frequency probes (above 15 MHz) has become increasingly important in musculoskeletal diagnostics, providing detailed images that allow for the rapid identification of iliopsoas bursitis within seconds.^10^ HRUS has also demonstrated utility beyond musculoskeletal applications, such as in breast imaging, where it offers excellent resolution for evaluating soft tissue structures.^11,12^ The development of standardized procedures, as outlined in the European Alliance of Associations for Rheumatology (EULAR) guidelines for ultrasound imaging in rheumatology, further supports the utility of ultrasound in musculoskeletal and extra-articular pathologies, ensuring consistency and reliability in diagnostic approaches.^13^

Magnetic resonance imaging (MRI), on the other hand, remains the gold standard for assessing BI due to its superior soft tissue contrast and ability to delineate size, shape, and communication of the bursa with the hip joint. It is particularly useful for identifying associated conditions, such as femoral head necrosis or synovial thickening.^14^

Computed tomography (CT) is another diagnostic tool that has been used successfully for BI, but it has significant limitations in soft tissue evaluation due to its lower contrast resolution compared to MRI. CT relies on X-ray attenuation differences, which are less distinct in soft tissues, making it challenging to identify subtle inflammation or to differentiate the bursa from surrounding structures.^2^ These limitations highlight the importance of selecting appropriate imaging modalities based on clinical presentation and the resources available.

Treatment of BI ranges from conservative treatment to operative interventions. Initial conservative strategies are rest, nonsteroidal anti-inflammatory medications and physiotherapy, aspiration of fluid, and steroid injections. For patients exhibiting persistent or severe symptoms, surgery may be considered.^2,15^ Complete removal of a symptomatic synovial cyst,^4^ debridement of the check-valve mechanism to the hip joint, and emptying the cyst by hip arthroscopy^15^ as well as removal of wear debris after THA have been reported to successfully alleviate symptoms.

Due to the rarity of BI and variability in its presentation, systematic reviews of case reports and series are essential to improve the understanding and to guide diagnosis and treatment. This review aims to analyze current research on BI to synthesize its clinical presentation, risk factors, diagnostic methods, treatment modalities, and outcomes.

## Materials and methods

### Search strategy

The search strategies were developed by considering the heterogeneous terminology for BI. Terminology was extracted from approximately 200 seed papers that were known to be relevant before the search. The searches were composed by an information specialist (CA-H) and reviewed by a second information specialist. The bibliographic databases Embase (embase.com), Medline (Ovid), and the Web of Science Core Collection (Clarivate Analytics) were searched using database-specific subject headings and text word synonyms covering BI, whereas the search syntax was translated from embase.com to Ovid and to Web of Science by publicly available macros.^16^

The last search was performed on 5 April 2022. No language or publication date restrictions were applied. Full-search strategies are documented in *Supplemental Digital Content 1*. All references were exported to EndNote 20 and deduplicated using the Bramer method.^17^

### Eligibility criteria

We included reports that (1) described a diagnosis of BI, (2) offered clear descriptions of clinical features, (3) provided sufficient detail to assess the diagnosis, and (4) were written in English. The exclusion criteria were (1) cases with insufficient information, (2) duplicate reports of the same case, (3) infectious etiology, (4) no confirmation with diagnostic imaging, and (5) cases published as abstracts only.

### Study selection

The titles and abstracts of the search results were screened by two authors (MLJ, YM). Selected references were obtained in full-text and independently assessed for eligibility by the same reviewers. Disagreements over eligibility were resolved by consensus or through author arbitration (AI or LB). Reference lists of eligible articles were checked for potential additional records that met the inclusion criteria.

### Data extraction and quality assessment

Data extraction from the included studies was carried out independently by two authors (MLJ, YM). The extracted and tabulated data included (1) patient characteristics, including age, gender, and medical history, type of arthropathy, and comorbidities, such as arthritis, avascular necrosis, synovial chondromatosis, pseudogout, and history of THA; (2) clinical features including location and duration of pain, reduction of mobility, presence of swelling or tenderness, compression symptoms, and any other associated symptoms or physical examination findings; (3) diagnostic methods included all utilized imaging techniques (conventional X-ray, US, CT, MRI); (4) treatment modalities ranged from medication and physical therapy to injection therapy, aspiration, surgical interventions (bursectomy, mass resection, total hip replacement); (5) outcomes assessed were pain score, symptoms relief, and recurrence rates. Any discrepancies were resolved through consensus or third-author arbitration (AI or LB).

### Quality assessment

We used a slightly modified version of the Joanna Briggs Institute tool to systematically assess the methodological quality and potential biases of the included studies.^18^ We used seven specific items from the tool: (1) Were the patient’s demographic characteristics clearly described? (2) Was the patient’s history clearly described and presented as a timeline? (3) Was the current clinical condition of the patient on presentation clearly described? (4) Were diagnostic tests or assessment methods and the results clearly described? (5) Was/were the intervention/s or treatment procedure/s clearly described? (6) Was the postintervention clinical condition clearly described? (7) Does the case report provide takeaway lessons? Ratings for each item were assigned as “yes,” “no,” “unclear,” or “not applicable” to accurately capture the comprehensiveness and clarity of the reported data.

### Statistical analysis

Missing data points were not imputed. All analyses were conducted based on the appropriate available case set. All analyses were performed using R version 4.3.2 (R Foundation for Statistical Computing, Vienna, Austria) (2023-10-31).

#### Associations between recurrence and treatment modalities

We tested for an association between treatment modality and recurrence rate using a multivariable generalized linear model with binomial distribution of the dependent variable (recurrence) and a logit link function and including all the treatment modalities. We also ran separate univariable models for each of the treatment modalities. In addition, we ran a generalized linear model using “prioritized” treatment modalities as a 9-level factor with “Rest” as the reference level to assess whether other treatments result in a higher or lower risk of recurrence compared to the least invasive treatment modality. For the latter model, due to rare or no cases of recurrence in one or several of the treatment modalities, we applied Firth’s penalization for rare events to compute meaningful 95% confidence intervals and *p*-values.

#### Associations between treatment modalities and efficacy

We explored the connection between treatment methods and pain relief by comparing pain levels before and after treatment, categorizing the changes as either no change/increase or decrease in pain, and used generalized linear models to analyze the data. Due to limited data on pre- and posttreatment pain levels, we did not construct a multivariable model, but applied Firth’s penalization to address rare events. Similarly, we examined treatment efficacy and risk factors for recurrence using generalized linear models, adjusting for the scarcity of events with Firth’s penalization as needed.

For all binary endpoints, we used marginal odds ratios because they allow comparison of the association between a dependent variable and a set of explanatory factors across different studies and datasets.^19^

Following the recommendations by the American Statistical Association,^20^ we did not use an arbitrary threshold of *p*-values (e.g., 0.05) to interpret our results. In contrast, we used *p*-values, effect sizes (e.g., odds-ratios), and 95% confidence intervals around the point estimates to assess the strength of the statistical evidence in favor of a weak, moderate, or strong association.

### Ethical review

Ethical approval was not required for this systematic review, as it is based on published studies.

## Results

### Identification of studies

**Figure 2.**
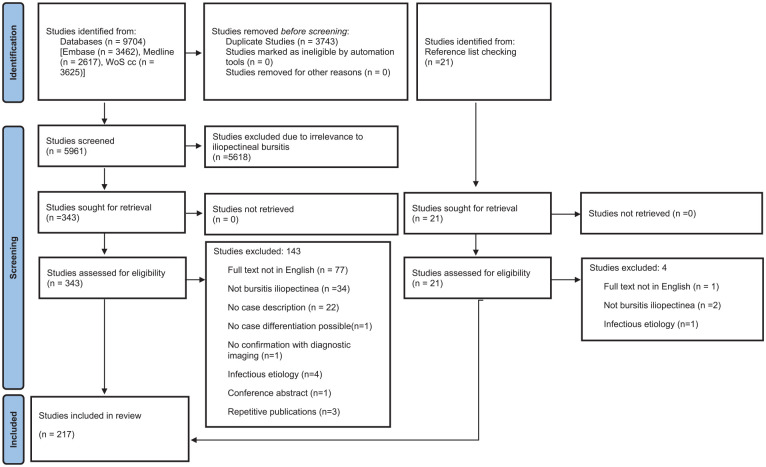
A detailed procedure of literature selection according to PRISMA guidelines.

The initial search yielded 5961 studies. After reviewing the titles and abstracts, 5618 studies were deemed ineligible because they did not address iliopectineal bursitis. The remaining 343 papers were selected for full-text review, of which 143 were excluded. A total of 21 additional publications were identified from cited references. From these 21 papers, 4 were excluded after eligibility review, resulting in 217 included studies covering 502 individual cases. The detailed process of literature selection is shown in Figure 2, according to PRISMA guidelines.^21^

### Quality assessment

The overall quality of the analyzed studies was good to moderate. Most case reports provided a clear description of patient demographics (98%), a timeline of the patient’s history (88%), a description of the patient’s clinical condition on presentation (97%), a clear description of diagnostic tests and assessment methods (99%), a description of intervention or treatment methods (89%), the patient’s postintervention clinical condition (67%), and takeaway lessons (80%). Detailed results as well as the reference list for the included studies are given in *Supplemental digital content 2*.

### Patient characteristics, comorbidities, and clinical outcomes

The mean patient age was 57.4 years. Sex and the affected side of the hip were found to be evenly distributed among patients. The most prevalent comorbidity was osteoarthritis, THR with a mean time after implantation of 2.5 years, and rheumatoid arthritis. An overview is given in Table 1.

**Table 1.** Patient characteristics, comorbidities and clinical outcomes. *N* = 502; numbers do not add up to 502% or 100%, respectively, due to missing information.

Characteristic	Count (%)
Number of patients	502
Sex	
Female	248 (50.1)
Male	247 (49.9)
Age (mean, SD)	57.4 (14.4)
Side	
Right	196 (48.4)
Left	209 (51.6)
Comorbidities	
Osteoarthritis	153 (30.5)
Total hip replacement	133 (26.5)
Years after implantation (median range)	2.5 (0.0–6.0)
Rheumatoid arthritis	90 (17.9)
Trauma	20 (4.0)
Femur head necrosis	16 (3.2)
Dysplasia	13 (2.6)
Sport induced bursitis	6 (1.2)
Polymyalgia rheumatic	4 (0.8)
Pseudogout	2 (0.4)
Systemic lupus erythematosus	2 (0.4)
Metastatic bone disease	1 (0.2)
Juvenile idiopathic arthritis	1 (0.2)
Clinical outcomes	
Recurrence after first-line treatment	107 (35.4)

SD: standard deviation.

### Terminology of BI and diagnostics imaging techniques

The most commonly used terms for BI were bursitis/enlarged bursa, tumor/mass, or cyst (see also Table 2).

**Table 2.** Different terms of Bursitis iliopectinea (BI) used in literature with their respective relative occurrence.

Lesion name	Count (%)
Bursitis/enlarged bursa	190 (37.8)
Tumor/mass	112 (22.3)
Cyst	99 (19.7)
Metallosis induced bursitis/pseudotumor	45 (9.0)
Ganglion	25 (5.0)
Granulomatous lesion	3 (0.6)

No single diagnostic method is universally applied, as demonstrated by the frequency and overlap of various techniques used in the study population (see also Table 3).

**Table 3.** Diagnostic techniques used in the literature in order to identify Bursitis iliopectinea (BI). *N* = 502; numbers add up to more than 502% or 100%, respectively, because in several cases more than one diagnostic technique was applied.

Diagnostic technique	Count (%)
X-ray	311 (62.0)
Ultrasound	217 (43.3)
CT (with or without contrast)	238 (47.4)
MRI (with or without contrast)	247 (49.2)

CT: computed tomography; MRI: magnetic resonance imaging.

### Correlation between comorbidities and symptoms

A LR test comparing two models with and without etiology-by-syndrome interaction found no correlation between comorbidities and syndromes (χ^2^ = 17.54, *df* = 30, *p* = 0.97). However, the additive model suggests that osteoarthritis and THR are more common than expected (*z* = 5.6, *p* < 0.001; *z* = 7.04, *p* < 0.001), along with musculoskeletal syndromes (*z* = 4.76, *p* < 0.001). Notably, vascular symptoms rank second for BI. An overview of these correlations can be seen in Table 4.

**Table 4.** Correlation between symptoms and comorbidities, including total hip replacement, identified as one of the etiologies. Of the 502 included cases, 208 with hip-related comorbidity presented a particular type of reported syndromes. Other common comorbidities were hip dysplasia, femur head necrosis, and osteoarthritis.

Comorbidity	*N*	Musculoskeletal	Neurological	Vascular
Dysplasia	13	5 (2.4)	0 (0)	2 (1)
Femur head necrosis	16	11 (5.3)	1 (0.5)	5 (2.4)
Osteoarthritis	153	54 (26)	7 (3.4)	30 (14.4)
Total hip replacement	133	107 (51.4)	22 (10.6)	51 (24.5)

In the analysis of the correlation between symptoms and comorbidities, in which THR was identified as one of the etiologies, 208 out of the 502 included cases with hip-related comorbidity exhibited a specific type of reported syndromes.

### Treatment modalities

The most common operative first-line treatments were the resection of the bursa (30%), total hip arthroplasty (29%), and aspiration of the bursa (24%). For conservative treatment, the use of analgesics (17%), injection of corticoids (11%), and physiotherapy (9%) was resorted to. In over 40% of patients, one or more treatments was used (see also Table 5).

**Table 5.** First-line treatment as postulated in the literature. Mass resection, total hip arthroplasty, and aspiration were most stated. Analgesic was used in only 17.51% of the cases but, however, may have been poorly reported. The initial therapy was anyway known in only 89% of the included cases. Nevertheless, numbers add up to more than 100% because in some cases more than one treatment was applied to the same patient.

Intervention (*N* = 474)	Count (%)
Mass resection	141 (29.75)
Total hip arthroplasty	137 (28.90)
Aspiration	112 (23.63)
Analgesic	83 (17.51)
Bursectomy	59 (12.45)
Corticosteroid injection	52 (10.97)
Physiotherapy	41 (8.65)
Rest	20 (4.22)
Systemic steroids	19 (4.01)
Immunosuppressant other than steroids	12 (2.53)

### Association between risk factors and compression syndromes

Nearly one-third of the patients suffered from compression syndromes, with compression of the femoral vein being the most frequent condition (16%). An overview of all registered compression syndromes can be seen in Table 6.

**Table 6.** Occurrence of compression symptoms in the literature. Deep vein thrombosis was included in this assessment, as it constitutes an indirect sign of compression.

Compression syndrome	Count (%)
Any compression syndrome	151 (30.1)
Compression of femoral vein	81 (16.1)
Compression of external iliac vein	39 (7.8)
Compression of femoral nerve	34 (6.8)
Compression of femoral artery	33 (6.6)
Deep vein thrombosis	14 (2.8)
Compression of external iliac artery	14 (2.8)
Compression of the ureter	6 (1.2)

In a multivariate model examining how different risk factors or comorbidities are associated with compression syndromes, only rheumatoid arthritis showed an association with the occurrence of compression syndromes (see also Table 7).

**Table 7.** Associations between risk factors and compression symptoms. There was a significant correlation of compression symptoms with the occurrence of rheumatoid arthritis (*p* = 0.011).

Comorbidity (*N* = 405)	Odds-ratio	OR 95% CI	Multivariable *p*-value
Metastatic bone disease	3.64	(0.158, 83.905)	0.420
Polymyalgia rheumatica	0.251	(0.014, 4.475)	0.347
Pseudogout	2.312	(0.255, 20.988)	0.457
Rheumatoid arthritis	1.873	(1.154, 3.04)	0.011
Sex	0.988	(0.656, 1.487)	0.953
Side of the BI = left (vs right)	1.176	(0.783, 1.767)	0.435
Systemic lupus erythematosus	1.578	(0.166, 15.025)	0.692
Sport-induced bursitis	0.175	(0.01, 3.059)	0.232
Trauma	0.701	(0.264, 1.859)	0.475

CI: confidence interval; BI: bursitis iliopectinea.

### Treatment modalities and clinical outcomes

#### Follow-up duration

Information regarding the follow-up times was given for 500 patients. Of those, 201 were re-examined at follow-up. The minimum and maximum follow-up times were 0.5 and 120 months, respectively, median follow-up time was 12 months (interquartile range 6–35).

#### Recurrence rates after first-line treatment

Information about recurrence of symptoms after first-line treatment was available for 302 patients. One hundred seven patients suffered from recurring symptoms, leading to an estimated 35.4% recurrence rate (95% CI: 30.07–41.03).

Symptoms appeared to recur more often when aspiration or physiotherapy were used. These associations were apparent both in the univariable and multivariable models. There is also some evidence that symptoms were more likely to recur after analgesics treatment with THR having the lowest probability of symptom recurrence. In Table 8, an overview of the symptoms is given.

**Table 8.** Estimated associations between symptom recurrence and first-line treatment modalities. A significant correlation was seen with use of analgesic (*p* = 0.034), aspiration (*p* < 0.001), bursectomy or mass resection (*p* = 0.043), physiotherapy (*p* = 0.003), total hip replacement (*p* = 0.013), and systemic steroids (*p* = 0.028). No significant correlation was seen in steroid injections and other immunosuppressant therapies.

First-line treatment	Marginal odds-ratio	Marginal odds-ratio 95% CI	*p*-Value
Analgesic	1.871	(1.049, 3.336)	0.034
Aspiration	4.468	(2.391, 8.35)	<0.001
Bursectomy/mass resection	0.529	(0.285, 0.981)	0.043
Immunosuppressant other than steroids	0.387	(0.092, 1.627)	0.195
Physiotherapy	4.102	(1.605, 10.487)	0.003
Total hip replacement	0.224	(0.069, 0.726)	0.013
Rest	0.345	(0.106, 1.121)	0.077
Steroid injection	0.647	(0.372, 1.127)	0.124
Systemic steroids	0.41	(0.185, 0.908)	0.028

CI: confidence interval.

None of the comorbidities explored was found to be associated with the risk of recurrence of BI after treatment.

## Discussion

BI is a rare condition closely related to pathologies of the hip joint. The terminology that is used for BI is heterogeneous and complicates the search for relevant literature. This is also reflected in the complex search strategies we had to apply for this systematic review. We opted for the term “BI,” as it reflects the accumulation of fluid in the bursa due to all possibly causes, such as osteoarthritis, mechanical irritation of the iliopsoas tendon, wear particles after THR, pseudogout, or rheumatoid arthritis. To our knowledge, this is the first systematic review on BI with a sufficient number of cases reviewed to draw conclusions about clinically relevant aspects, such as risk factors, etiologies, treatment outcomes, and recurrence rates.

Every systematic review has inherent limitations. The quality of the review is strongly dependent on the quality of the included studies. In addition, variability in study design can make it difficult to synthesize studies and draw definitive conclusions. We systematically assessed the methodological quality and potential biases of the included studies to ensure robust conclusions. The overall quality was good to moderate, with most studies clearly reporting patient demographics, clinical evaluation of the patient, description of diagnostic test and treatment methods as well as the postintervention course. The criteria for inclusion or exclusion of a study can have a significant impact on the conclusion of a systematic review. We have performed a comprehensive search to find all relevant studies, despite the heterogeneity of the terminology used for BI. The final selection of studies included in this review was based on the predetermined inclusion and exclusion criteria.

The diagnosis of BI can be challenging, and multiple imaging modalities are usually required to distinguish the condition from other inguinal masses.^1^ Ultrasound is the most used first-choice imaging technology due to its low costs, availability, and lack of radiation exposure. CT and MRI provide more detailed information regarding the characteristics of the bursa, connection with the hip joint, joint degeneration, and the presence of septa or synovial thickenings.^1^ Accordingly, we found that these three imaging techniques are the ones most often used to diagnose BI. Based on the study by Wunderbaldinger et al.,^14^ imaging for suspected BI should start with cost-effective ultrasound as the initial diagnostic tool. However, MRI is recommended for accurate assessment of bursa size, communication with the hip joint, and therapy planning due to its superior diagnostic accuracy.

Compression syndromes of the inguinal neurovascular structures are among the more serious consequences of BI. Patients in an early stage of the disease usually present with a local swelling and pain in the groin. Our results indicate that nearly a third (30.1%) of patients with BI develop compression syndromes of some kind, with compression of the femoral vein being the most common, constituting about half of the cases. Compression of the femoral vein can result in venous stasis, edema, and femoral vein thrombosis. Of those, deep vein thrombosis is particularly dangerous since it can be fatal.^22^ Neuropathy due to the compression of the femoral nerve following BI was previously discussed as being extremely rare.^6^ We found that 6.8% of the reported cases presented with neuropathy, which is a significant number and should therefore be considered when diagnosing BI.^22^

So far, treatment modalities for BI have largely been reported and discussed in case reports.^15^ Some authors have reviewed a fraction of these case reports focusing on special aspects of the condition. Colasanti et al.^23^ reviewed 27 cases, 19 of which were treated with a surgical excision and eight of which received a needle aspiration. Three patients that received needle aspiration showed recurrence, whereas only one of the 19 patients who were treated with surgical excision showed recurring symptoms. Lin et al.^3^ described an institutional cohort of 15 patients, 14 of which had undergone surgical resection. None of them showed recurrence. In contrast, the one patient that received needle aspiration developed recurrent symptoms within 1 month. Neither clinical nor radiological evidence of recurrence was also reported of a cohort of 30 patients who received surgical treatment by arthroscopic techniques.^2^ Our findings from 302 reviewed cases confirm that surgery addressing hip pathology was the most successful treatment of BI, while treatments such as aspiration, analgesics, or physiotherapy had significantly higher recurrence rates.

This study has certain limitations, including reporting biases favoring severe cases and variability in diagnostic criteria and nomenclature across studies. Many reports lacked standardized outcome measures, making direct comparisons of treatment effectiveness challenging. The retrospective nature of the data introduces further potential inaccuracies. Additionally, the lack of data on asymptomatic or conservatively managed cases limits the understanding of the natural history of BI.

## Conclusions

In conclusion, in patients with hip arthritis or after total hip arthroplasty that present with unspecific groin pain or signs of compression of the inguinal neurovascular structures, the possibility of an enlarged iliopsoas bursa should be evaluated with ultrasound and/or MRI. Depending on the cause of the bursitis, successful treatment consists of surgical resection of the bursa, total hip arthroplasty, or revision hip arthroplasty.

Despite these challenges outlined above, our review highlights several critical points. BI is a rare but significant condition often associated with hip arthritis or complications following total hip arthroplasty. It can present with neurovascular compression syndromes, necessitating careful clinical evaluation and appropriate imaging. Surgical interventions, particularly those addressing the underlying hip pathology, are the most effective treatment options with the lowest recurrence rates. There is a clear need for further prospective research to standardize diagnostic criteria, improve nomenclature consistency, and develop evidence-based management strategies.

Clinicians should maintain a heightened level of suspicion for BI in patients presenting with unexplained groin pain, hip arthritis, or neurovascular symptoms. Early diagnosis and targeted intervention are key to improving outcomes for these patients.

## Supplemental Material

sj-docx-1-smo-10.1177_20503121251317899 – Supplemental material for Epidemiology, clinical manifestation, diagnosis,and treatment of bursitis iliopectinea: A systematic review Supplemental material, sj-docx-1-smo-10.1177_20503121251317899 for Epidemiology, clinical manifestation, diagnosis,and treatment of bursitis iliopectinea: A systematic review by Malgorzata Lea Jonczy, Lorenz Büchler, Yadusha Mahenthiran, Fabrice Helfenstein, Christian Appenzeller-Herzog and Andrej Isaak in SAGE Open Medicine

sj-docx-2-smo-10.1177_20503121251317899 – Supplemental material for Epidemiology, clinical manifestation, diagnosis,and treatment of bursitis iliopectinea: A systematic review Supplemental material, sj-docx-2-smo-10.1177_20503121251317899 for Epidemiology, clinical manifestation, diagnosis,and treatment of bursitis iliopectinea: A systematic review by Malgorzata Lea Jonczy, Lorenz Büchler, Yadusha Mahenthiran, Fabrice Helfenstein, Christian Appenzeller-Herzog and Andrej Isaak in SAGE Open Medicine
