# Supplementary material for: Epidemiology, clinical manifestation, diagnosis,and treatment of bursitis iliopectinea: A systematic review
Source: SAGE Open Med. 2025 Feb 3;13:20503121251317899. doi: 10.1177/20503121251317899 (PMC11789115; doi:10.1177/20503121251317899)
Supplement: sj-docx-1-smo-10.1177_20503121251317899 – Supplemental material for Epidemiology, clinical manifestation, diagnosis,and treatment of bursitis iliopectinea: A systematic review [file sj-docx-1-smo-10.1177_20503121251317899.docx]

**Supplemental Digital Content 1: Search strategies**

*Embase.com*

(20220405; 3462 hits)

('iliopsoas bursitis'/de OR (((iliopectinea* OR ilio-pectinea* OR ileopectinea* OR ileo-pectinea* OR iliopsoas OR ilioposoas OR ilio-posoas OR ileopsoas OR psoas) NEAR/3 (bursitis OR 'inflamed bursa' OR 'irritated bursa' OR cyst OR cysts OR ganglion OR synovitis)) OR ((enlarge* OR large OR hypertroph* OR swell* OR extensi* OR disten* OR giant OR huge OR supernumerary OR synovitis) NEAR/6 (iliopectinea* OR ilio-pectinea* OR ileopectinea* OR ileo-pectinea* OR iliopsoas OR ilioposoas OR ilio-posoas OR ileopsoas OR psoas OR pectineus) NEAR/3 bursa*) OR ((intrapelvic OR intra-pelvic) NEXT/1 (mass or cyst))):ab,ti) OR

(('synovial cyst'/de OR 'bursitis'/de OR 'pseudotumor'/de OR 'ganglion cyst'/de OR (pseudotumour* OR pseudotumor* OR pseudoabscess OR pseudo-abscess OR ((synovial OR bursal) NEXT/1 (cyst OR cysts)) OR ganglion OR granuloma OR chondromatosis):ab,ti) AND ('hip'/de OR 'iliopsoas muscle'/exp OR 'acetabulum'/de OR 'iliac bone'/de OR 'pelvis'/de OR 'inguinal region'/exp OR 'hip disease'/de OR 'hip pain'/de OR (hip OR pelvis OR pelvic OR intrapelvic OR coxa OR coxal OR groin OR inguinal OR psoas OR acetabular OR juxtaacetabular OR acetabulum OR pectineus OR femoral OR iliac OR ilium OR iliopectinea* OR ilio-pectinea* OR ileopectinea* OR ileo-pectinea* OR iliopsoas OR ilioposoas OR ilio-posoas OR ileopsoas):ab,ti))

NOT

(('animal'/de OR 'animal experiment'/exp OR 'nonhuman'/de) NOT ('human'/exp OR 'human experiment'/de))

NOT

[conference abstract]/lim

*Medline (Ovid)*

(20220405; Ovid MEDLINE(R) ALL 1946 to April 04, 2022; 2617 hits)

(((iliopectinea* OR ilio-pectinea* OR ileopectinea* OR ileo-pectinea* OR iliopsoas OR ilioposoas OR ilio-posoas OR ileopsoas OR psoas) ADJ3 (bursitis OR inflamed bursa OR irritated bursa OR cyst OR cysts OR ganglion OR synovitis))OR ((enlarge* OR large OR hypertroph* OR swell* OR extensi* OR disten* OR giant OR huge OR supernumeraryOR synovitis) ADJ6 (iliopectinea* OR ilio-pectinea* OR ileopectinea* OR ileo-pectinea* OR iliopsoas OR ilioposoasOR ilio-posoas OR ileopsoas OR psoas OR pectineus) ADJ3 bursa*) OR ((intrapelvic OR intra-pelvic) ADJ (mass or cyst))).ab,ti. OR

((synovial cyst/ OR bursitis/ OR ganglion cysts/ OR (pseudotumour* OR pseudotumor* OR pseudoabscess OR pseudo-abscess OR ((synovial OR bursal) ADJ (cyst OR cysts)) OR ganglion OR granuloma OR chondromatosis).ab,ti.) AND (hip/ OR hip joint/ OR pelvic bones/ OR acetabulum/ OR ilium/ OR groin/ OR hip injuries/OR (hip OR pelvis OR pelvic OR intrapelvic OR coxa ORcoxal OR groin OR inguinal OR psoas OR acetabular OR juxtaacetabular OR acetabulum OR pectineus OR femoral OR iliac OR ilium OR iliopectinea* OR ilio-pectinea* OR ileopectinea* OR ileo-pectinea* OR iliopsoas OR ilioposoasOR ilio-posoas OR ileopsoas).ab,ti.))

NOT (exp animals/ NOT humans/)

*Web of Science Core Collection*

(20220405; Editions = A&HCI , BKCI-SSH , BKCI-S , CCR-EXPANDED , ESCI , IC , CPCI-SSH , CPCI-S , SCI-EXPANDED , SSCI; 3625 hits)

TS=((((iliopectinea* OR ilio-pectinea* OR ileopectinea* OR ileo-pectinea* OR iliopsoas OR ilioposoas OR ilio-posoas OR ileopsoas OR psoas) NEAR/2 (bursitis OR "inflamed bursa"OR "irritated bursa" OR cyst OR cysts OR ganglion OR synovitis)) OR ((enlarge* OR large OR hypertroph* OR swell* OR extensi* OR disten* OR giant OR huge OR supernumerary OR synovitis) NEAR/6 (iliopectinea* OR ilio-pectinea* OR ileopectinea* OR ileo-pectinea* OR iliopsoas OR ilioposoas OR ilio-posoas OR ileopsoas OR psoas OR pectineus) NEAR/2 bursa*) OR ((intrapelvic OR intra-pelvic) NEAR/1 (mass or cyst)) OR ((pseudotumour* OR pseudotumor* OR pseudoabscess OR pseudo-abscess OR ((synovial OR bursal) NEAR/1 (cyst OR cysts)) OR ganglion OR granuloma OR chondromatosis) AND (hip OR pelvis OR pelvic OR intrapelvic OR coxa OR coxal OR groin OR inguinal OR psoas OR acetabular OR juxtaacetabular OR acetabulum OR pectineus OR femoral OR iliac OR ilium OR iliopectinea* OR ilio-pectinea* OR ileopectinea* OR ileo-pectinea* OR iliopsoas OR ilioposoas OR ilio-posoas OR ileopsoas))))
