# Supplementary material for: Epidemiology, clinical manifestation, diagnosis,and treatment of bursitis iliopectinea: A systematic review
Source: SAGE Open Med. 2025 Feb 3;13:20503121251317899. doi: 10.1177/20503121251317899 (PMC11789115; doi:10.1177/20503121251317899)
Supplement: sj-docx-2-smo-10.1177_20503121251317899 – Supplemental material for Epidemiology, clinical manifestation, diagnosis,and treatment of bursitis iliopectinea: A systematic review [file sj-docx-2-smo-10.1177_20503121251317899.docx]

**Supplemental Digital Content 2: Quality assessment**

Quality assessment of the reports included in this review

| **no** | **Author** | **Year** | **Country** | **Were patient´s demographic characteristics clearly described?** | **Was the patient´s history clearly described and presented as a timeline?** | **Was the current clinical condition of the patient on presentation clearly described?** | **Were diagnostic tests or assessment methods and the results clearly described?** | **Was the intervention(s) or treatment procedure(s) clearly described?** | **Was the post-intervention clinical condition clearly described?** | **Does the case report provide takeaway lessons?** |
| --- | --- | --- | --- | --- | --- | --- | --- | --- | --- | --- |
| 1 | Albaba [1] | 2008 | USA | Yes | Yes | Yes | Unclear | Yes | No | Yes |
| 2 | Algarni et al. [2] | 2012 | Canada | Yes | Yes | Yes | Yes | Yes | Yes | Yes |
| 3 | Al-Kodairy et al. [3] | 1997 | Switzerland | Yes | Yes | Yes | Yes | Yes | Yes | Yes |
| 4 | Alter et al. [4] | 2021 | USA | Yes | Yes | Yes | Yes | Yes | Yes | Yes |
| 5 | Angelini et al. [5] | 2017 | Italy | Yes | Yes | Yes | Yes | Yes | Yes | Yes |
| 6 | Armstrong and Saxton [6] | 1972 | UK | Yes | Unclear | Yes | Yes | Yes | Yes | Yes |
| 7 | Atkinson [7] | 1986 | Canada | Yes | Unclear | Yes | Yes | Yes | Yes | Yes |
| 8 | Avasarala and Absan [8] | 2016 | USA | Yes | Yes | Yes | Yes | Yes | Yes | Yes |
| 9 | Bakx and Wiggers [9] | 1996 | The Netherlands | Yes | Yes | Yes | Yes | Yes | No | Yes |
| 10 | Beardsmore et al. [10] | 2000 | UK | Yes | Yes | Yes | Yes | Yes | Unclear | No |
| 11 | Bekou et al. [11] | 2011 | Switzerland | Yes | Yes | Yes | Yes | Yes | Yes | Yes |
| 12 | Beksaç et al. [12] | 2007 | Turkey | Yes | Yes | Yes | Yes | Yes | Yes | Yes |
| 13 | Bergenudd et al. [13] | 1987 | Sweden | Yes | Yes | Yes | Yes | Yes | Yes | Yes |
| 14 | Bhargava et al. [14] | 2004 | UK | Yes | Yes | Yes | Yes | Yes | Unclear | Yes |
| 15 | Bianchi et al. [15] | 2002 | Switzerland | Yes | Yes | Yes | Yes | No | No | Yes |
| 16 | Bilora et al. [16] | 2017 | Italy | Yes | Yes | Yes | Yes | Yes | Yes | No |
| 17 | Binek and Levinsohn [17] | 1987 | USA | Yes | Yes | Yes | Yes | Yes | Yes | Yes |
| 18 | Bobrowski et al. [18] | 2018 | Germny | Yes | Yes | Yes | Yes | Yes | Yes | Yes |
| 19 | Bolhuis et al. [19] | 1990 | The Netherlands | Yes | Yes | Yes | Yes | Yes | Yes | Yes |
| 20 | Brunot et al. [20] | 2013 | France | Yes | Yes | Yes | Yes | Yes | No | Yes |
| 21 | Burnett et al. [21] | 2018 | USA | Yes | Yes | Yes | Yes | Yes | Yes | Yes |
| 22 | Butler and Barrack [22] | 2004 | USA | Yes | Yes | Yes | Yes | Yes | Yes | Yes |
| 23 | Byrne et al. [23] | 1996 | UK | Yes | Yes | Yes | Yes | No | No | Yes |
| 24 | Byström et al. [24] | 1995 | Sweden | Yes | Yes | Yes | Yes | Yes | Yes | Yes |
| 25 | Cantini et al. [25] | 1999 | Italy | Yes | Yes | Yes | Yes | Yes | Unclear | No |
| 26 | Cassina et al. [26] | 2000 | Switzerland | Yes | Yes | Yes | Yes | No | No | Yes |
| 27 | Chaiamnuay and Davis [27] | 1984 | Canada | No | No | Yes | Yes | No | No | No |
| 28 | Chalmers and Chalmers [28] | 1992 | UK | Yes | Yes | Yes | Yes | Yes | Yes | Yes |
| 29 | Chen and Tsai [29] | 2017 | Taiwan | Yes | Yes | Yes | Yes | Unclear | Unclear | Yes |
| 30 | Cheung et al. [30] | 2004 | UK | Yes | Yes | Yes | Yes | Yes | Yes | Yes |
| 31 | Chilton and Darke [31] | 1980 | UK | Yes | Yes | Yes | Yes | Yes | Yes | No |
| 32 | Cho et al. [32] | 2019 | Republic of Korea | Yes | Yes | Yes | Yes | Yes | Yes | Yes |
| 33 | Chong et al. [33] | 2015 | Republic of Korea | Yes | Yes | Yes | Yes | Yes | Yes | Yes |
| 34 | Clarke et al. [34] | 1999 | UK | Yes | Yes | Yes | Yes | Yes | Yes | Yes |
| 35 | Cohen et al. [35] | 1985 | USA | Yes | Yes | Yes | Yes | Unclear | No | Yes |
| 36 | Colasanti et al. [36] | 2006 | Italy | Yes | Yes | Yes | Yes | Yes | Yes | Yes |
| 37 | Conroy et al. [37] | 2021 | USA | Yes | Yes | Yes | Yes | Yes | Yes | Yes |
| 38 | Cook [38] | 1952 | USA | Yes | Yes | Yes | Yes | Yes | Unclear | No |
| 39 | Corvino et al. [39] | 2020 | Italy | Yes | Yes | Yes | Yes | Yes | Yes | Yes |
| 40 | Coulier and Cloots [40] | 2003 | Belgium | Yes | Yes | Yes | Yes | Unclear | No | No |
| 41 | Coventry et al. [41] | 1959 | USA | Yes | Yes | Yes | Yes | Yes | Yes | Yes |
| 42 | Czuszman et al. [42] | 2017 | USA | Yes | Yes | Yes | Yes | Unclear | No | Yes |
| 43 | Dan et al. [43] | 2021 | Japan | Yes | Not applicable | Not Applicable | Yes | No | No | Yes |
| 44 | DeFrancesco and Kamath [44] | 2015 | USA | Yes | Yes | Yes | Yes | Yes | Yes | Yes |
| 45 | DeFrang et al. [45] | 1996 | USA | Yes | Yes | Yes | Yes | Yes | Yes | Yes |
| 46 | Di Benedetto et al. [46] | 2019 | Italy | Yes | Yes | Yes | Yes | Yes | Yes | Yes |
| 47 | Di Carlo et al. [47] | 2015 | Italy | Yes | Yes | Yes | Yes | Unclear | Unclear | Yes |
| 48 | Di Sante et al. [48] | 2014 | Italy | Yes | Yes | Yes | Yes | Yes | Yes | Yes |
| 49 | DiMaio and Santore [49] | 1997 | USA | Yes | Yes | Yes | Yes | Yes | Yes | No |
| 50 | Emery and Griffiths [50] | 1997 | UK | Yes | Yes | Yes | Yes | Yes | Yes | Yes |
| 51 | Emura et al. [51] | 2005 | Japan | Yes | Yes | Yes | Yes | Yes | Yes | Yes |
| 52 | Endo et al. [52] | 1990 | Japan | Yes | Yes | Yes | Yes | Yes | No | Yes |
| 53 | Enzler et al. [53] | 2000 | Switzerland | Yes | Yes | Yes | Yes | Yes | Yes | Yes |
| 54 | Farrington et al. [54] | 2002 | UK | Yes | Yes | Yes | Yes | Yes | Yes | Yes |
| 55 | Finder [55] | 1938 | USA | Yes | Unclear | Yes | Yes | Yes | Unclear | Unclear |
| 56 | Flanagan et al. [56] | 1995 | Ireland | Yes | Yes | Yes | Yes | Yes | Yes | Yes |
| 57 | Fokter et al. [57] | 2009 | Slovenia | Yes | Yes | Yes | Yes | Yes | Yes | Yes |
| 58 | Ford et al. [58] | 1981 | UK | Yes | Unclear | Yes | Yes | Yes | Yes | Yes |
| 59 | Forster et al. [59] | 1989 | Canada | Yes | Yes | Yes | Yes | Yes | Yes | Yes |
| 60 | Fortin and Bélanger [60] | 1995 | Canada | Yes | Yes | Yes | Yes | Yes | Yes | Yes |
| 61 | Fukui et al. [61] | 2019 | Japan | Yes | Yes | Yes | Yes | Yes | Yes | Yes |
| 62 | Fukui et al. [62] | 2015 | Japan | Yes | Yes | Yes | Yes | Yes | Yes | Yes |
| 63 | Gale et al. [63] | 1990 | USA | Yes | Yes | Yes | Yes | Yes | Yes | No |
| 64 | Gatch and Green [64] | 1925 | USA | Yes | Yes | Yes | Yes | Yes | Yes | Yes |
| 65 | Generini and Matucci-Cerinic [65] | 1993 | Italy | Yes | Yes | Yes | Yes | Yes | Yes | Yes |
| 66 | Ginesty et al. [66] | 1998 | France | Yes | No | Yes | Yes | Unclear | Yes | Yes |
| 67 | Gömöri et al. [67] | 2021 | Hungary | Yes | Yes | Yes | Yes | Yes | Yes | Yes |
| 68 | Gong et al. [68] | 2010 | China | Yes | Yes | Yes | Yes | Unclear | Yes | Yes |
| 69 | Goupille et al. [69] | 1990 | France | Yes | Yes | Yes | Yes | Not applicable | Not applicable | Yes |
| 70 | Grindulis et al. [70] | 1982 | UK | Yes | Yes | Yes | Yes | Yes | Yes | Yes |
| 71 | Gruber et al. [71] | 2007 | Austria | Yes | Yes | Yes | Yes | Yes | Yes | Yes |
| 72 | Harris et al. [72] | 1987 | USA | Yes | Yes | Yes | Yes | Yes | Yes | Yes |
| 73 | Harris et al. [73] | 1997 | USA | Yes | Yes | Yes | Yes | Yes | Yes | Yes |
| 74 | Hauptfleisch et al. [74] | 2011 | UK | Yes | Unclear | Yes | Yes | Unclear | Unclear | Yes |
| 75 | Helfgott [75] | 1988 | USA | Yes | Yes | Yes | Yes | Yes | Yes | Yes |
| 76 | Holton et al. [76] | 2014 | UK | Yes | Unclear | Yes | Yes | Yes | No | No |
| 77 | Howie et al. [77] | 1991 | Australia | Yes | No | No | Unclear | No | No | Yes |
| 78 | Hung et al. [78] | 2016 | Taiwan | Yes | Yes | Yes | Yes | Yes | Yes | Yes |
| 79 | Huang et al. [79] | 2014 | Taiwan | Yes | Yes | Yes | Yes | Yes | Yes | No |
| 80 | Huang et al. [80] | 2010 | Taiwan | Yes | Yes | Yes | Yes | Unclear | Unclear | Yes |
| 81 | Ikard [81] | 1991 | USA | Yes | Yes | Yes | Yes | Yes | Yes | Yes |
| 82 | Iversen et al. [82] | 1996 | Denmark | Yes | Yes | Yes | Yes | Yes | Yes | Yes |
| 83 | Iwata et al. [83] | 2013 | Japan | Yes | Yes | Yes | Yes | Yes | Yes | Yes |
| 84 | Janus and Hermann [84] | 1982 | USA | Yes | Yes | Yes | Yes | Yes | No | Yes |
| 85 | Jeremy [85] | 1969 | Australia | Yes | Yes | Yes | Yes | Yes | Yes | No |
| 86 | Jerosch et al. [86] | 2021 | Germany and Egypt | Yes | Yes | Yes | Yes | Yes | Yes | Yes |
| 87 | Jones et al. [87] | 1993 | UK | Yes | Yes | Yes | Yes | Yes | Yes | Yes |
| 88 | Jung et al. [88] | 2019 | Republic of Korea | Yes | Yes | Yes | Yes | Yes | No | Yes |
| 89 | Kalaci et al. [89] | 2009 | Turkey | Yes | Yes | Yes | Yes | Yes | Yes | Yes |
| 90 | Kanachi et al. [90] | 2014 | Japan | Yes | Yes | Yes | Yes | Yes | Yes | Yes |
| 91 | Kataoka et al. [91] | 1995 | Japan | Yes | Yes | Yes | Yes | Yes | No | Yes |
| 92 | Kawakita et al. [92] | 2013 | Japan | Yes | Yes | Yes | Yes | Yes | No | Yes |
| 93 | Kawasaki et al. [93] | 2013 | Yes | Yes | Yes | Yes | Yes | Yes | Yes | Yes |
| 94 | Keese et al. [94] | 2017 | Germany | Yes | Yes | Yes | Yes | Yes | Yes | Yes |
| 95 | Kenaan et al. [95] | 1999 | UK | Yes | Yes | Yes | Yes | Yes | Yes | Yes |
| 96 | Kerry et al. [96] | 2000 | UK | Yes | Yes | Yes | Yes | Yes | Yes | Yes |
| 97 | Kim et al. [97] | 2020 | Republic of Korea | Yes | Yes | Yes | Yes | Yes | Yes | Yes |
| 98 | Kim and Cho [98] | 2014 | Republic of Korea | Yes | Yes | Yes | Yes | Yes | Yes | Yes |
| 99 | Kim et al. [99] | 2016 | Republic of Korea | Yes | Yes | Yes | Yes | Yes | Yes | No |
| 100 | Kolmert et al. [100] | 1984 | Sweden | Yes | Yes | Yes | Yes | Yes | Yes | Yes |
| 101 | Kosuge et al. [101] | 2007 | UK | Yes | Yes | Yes | Yes | Yes | No | Yes |
| 102 | Kozlov ans Sonin [102] | 1998 | USA | Yes | No | Yes | Yes | Yes | Yes | Yes |
| 103 | Kuroyanagi et al. [103] | 2013 | Japan | Yes | Yes | Yes | Yes | Yes | Yes | Yes |
| 104 | Kurze et al. [104] | 2014 | Switzerland | Yes | Yes | Yes | Yes | Yes | Yes | Yes |
| 105 | Lavyne et al. [105] | 1982 | USA | Yes | Yes | Yes | Yes | Yes | Yes | Yes |
| 106 | Lax Pérez et al. [106] | 2012 | Spain | Yes | Yes | Yes | Yes | Yes | Yes | Yes |
| 107 | Leekam et al. [107] | 1985 | Canada | Yes | Yes | Yes | Yes | Yes | No | Yes |
| 108 | Létourneau et al. [108] | 1991 | Canada | Yes | Yes | Yes | Yes | Yes | Yes | No |
| 109 | Leung and Kudrna [109] | 2016 | USA | Yes | Yes | Yes | Yes | Yes | Yes | Yes |
| 110 | Levy et al. [110] | 1982 | USA | Yes | Yes | Yes | Yes | Yes | Yes | Yes |
| 111 | Li et al. [111] | 2017 | China | Yes | Yes | Yes | Yes | Yes | Unclear | Yes |
| 112 | Lim et al. [112] | 2003 | Australia | Yes | Yes | Yes | Yes | Yes | Yes | Yes |
| 113 | Liman et al. [113] | 2011 | Germany | Yes | Yes | Yes | Yes | Yes | Yes | Yes |
| 114 | Lin et al. [114] | 2002 | Taiwan | Yes | Yes | Yes | Yes | Yes | No | Yes |
| 115 | Lin et al. [115] | 2001 | Taiwan | Yes | Yes | Yes | Yes | Yes | Yes | Yes |
| 116 | Loneragan et al. [116] | 1994 | Australia | Yes | No | Yes | Yes | Yes | No | Yes |
| 117 | Lupetin and Daffner [117] | 1990 | USA | Yes | Yes | Yes | Yes | Yes | Unclear | Yes |
| 118 | Mährlein et al. [118] | 2001 | Germany | Yes | Yes | Yes | Yes | Yes | Unclear | Yes |
| 119 | Matsumoto et al. [119] | 1992 | Japan | Yes | Yes | Yes | Yes | Yes | Unclear | No |
| 120 | Matsumoto et al. [120] | 2012 | Japan | Yes | No | Yes | Yes | Yes | Yes | Yes |
| 121 | Matsumoto et al. [121] | 2006 | Japan | Yes | Yes | Yes | Yes | Yes | Yes | No |
| 122 | Maurer et al. [122] | 2011 | Austria | Yes | Yes | Yes | Yes | Yes | Yes | Yes |
| 123 | McGraw et al. [123] | 1991 | Canada | Yes | Yes | Yes | Yes | Yes | Yes | Yes |
| 124 | McLaughlin [124] | 2002 | USA | Yes | Yes | Yes | Yes | Yes | Yes | Yes |
| 125 | Meaney et al. [125] | 1992 | UK | Yes | Yes | Yes | Yes | Unclear | Unclear | Yes |
| 126 | Melamed et al. [126] | 1967 | USA | Yes | Yes | Yes | Yes | Yes | No | Yes |
| 127 | Monaghan et al. [127] | 2014 | USA | Yes | Yes | Yes | Yes | Yes | Yes | Yes |
| 128 | Morales-Gonzáles et al. [128] | 2005 | Spain | Yes | Yes | Yes | Yes | Yes | Unclear | Yes |
| 129 | Mori et al. [129] | 2004 | Japan | Yes | Yes | Yes | Yes | Yes | Yes | Yes |
| 130 | Morita et al. [130] | 1997 | Japan | Yes | Yes | Yes | Yes | Yes | No | No |
| 131 | Murphy et al. [131] | 2010 | Ireland | Yes | Yes | Yes | Yes | Yes | Unclear | Yes |
| 132 | Nasra et al. [132] | 2021 | USA | Yes | Yes | Yes | Yes | Yes | Yes | Yes |
| 133 | Natsume et al. [133] | 2015 | Japan | Yes | Yes | Yes | Yes | Yes | Yes | Yes |
| 134 | Nazarian and Zeni [134] | 2012 | USA | Yes | Yes | Yes | Yes | Yes | Yes | Yes |
| 135 | Nihal and Drabu [135] | 1998 | UK | Yes | No | Yes | Yes | Yes | No | Yes |
| 136 | O´Connor [136] | 1933 | USA | Yes | Yes | Yes | Yes | Yes | Yes | Yes |
| 137 | O´Riordan et al. [137] | 2002 | UK | Yes | Yes | Yes | Yes | Yes | Unclear | Yes |
| 138 | Ornetti et al. [138] | 2010 | France | Yes | Yes | Yes | Yes | Yes | No | No |
| 139 | Oshima et al. [139] | 2018 | Japan | Yes | Yes | Yes | Yes | Yes | Yes | No |
| 140 | Oshima et al. [140] | 2021 | Japan | Yes | Yes | Yes | Yes | Yes | Unclear | No |
| 141 | Pachore et al. [141] | 2019 | India | Yes | Yes | Yes | Yes | Yes | Yes | No |
| 142 | Pandit et al. [142] | 2008 | UK | Yes | Yes | Yes | Yes | Yes | Yes | Yes |
| 143 | Parfitt et al. [143] | 2012 | UK | Yes | Yes | Yes | Yes | Yes | Yes | Yes |
| 144 | Park et al. [144] | 2009 | Republic of Korea | Yes | Yes | Yes | Yes | Yes | Yes | Yes |
| 145 | Parziale et al. [145] | 2009 | USA | Yes | Unclear | Yes | Yes | Yes | Yes | Yes |
| 146 | Patkat et al. [146] | 1999 | India | Yes | Yes | Yes | Yes | Yes | No | Yes |
| 147 | Pellman et al. [147] | 1986 | USA | Yes | Yes | Yes | Yes | Yes | Unclear | Unclear |
| 148 | Penkawa [148] | 1980 | USA | Yes | Unclear | Yes | Yes | Yes | Unclear | No |
| 149 | Persson et al. [149] | 2018 | Sweden | Yes | Yes | Yes | Yes | Yes | Unclear | No |
| 150 | Peters et al. [150] | 1980 | USA | Yes | Yes | Yes | Yes | Unclear | No | Yes |
| 151 | Pritchard et al. [151] | 1990 | USA | Yes | Yes | Yes | Yes | No | No | Yes |
| 152 | Ramage and Brown Morton [152] | 1934 | UK | Yes | Yes | Yes | Yes | Yes | Unclear | No |
| 153 | Ramsay and Donnelly [153] | 1999 | Australia | Yes | Yes | Yes | Yes | Yes | No | No |
| 154 | Raymond and Christiansen [154] | 2020 | USA | Yes | Yes | Yes | Yes | Yes | Yes | No |
| 155 | Regis et al. [155] | 2008 | Italy | Yes | Yes | Yes | Yes | Yes | Yes | Unclear |
| 156 | Ricci and Özcakar [156] | 2019 | Italy | Yes | Yes | Yes | Yes | Yes | Yes | Yes |
| 157 | Robinson et al. [157] | 2007 | UK | Yes | Yes | Yes | Yes | Yes | Yes | No |
| 158 | Rodriguez-Gomez et al. [158] | 2004 | Spain | Yes | Yes | Yes | Yes | Yes | Yes | Yes |
| 159 | Salmerón et al. [159] | 1999 | Spain | Yes | Yes | Yes | Yes | Yes | No | Yes |
| 160 | Samuelson et al. [160] | 1971 | USA | Yes | Yes | Yes | Yes | Yes | No | Yes |
| 161 | Saraiva et al. [161] | 2021 | Portugal | Yes | No | Yes | Yes | Yes | No | Yes |
| 162 | Sartoris et al. [162] | 1985 | USA | Yes | Yes | Yes | Yes | No | No | Yes |
| 163 | Savarese et al. [163] | 1991 | USA | Yes | Yes | Yes | Yes | Yes | Yes | Yes |
| 164 | Schnarkowski et al. [164] | 1996 | USA | Yes | No | No | Yes | Unclear | No | Yes |
| 165 | Seo et al. [165] | 2018 | Republic of Korea | Yes | Yes | Yes | Yes | Yes | Yes | Yes |
| 166 | Hwang et al. [166] | 2006 | Republic of Korea | Yes | Yes | Yes | Yes | Yes | Yes | Yes |
| 167 | Shimbo et al. [167] | 2021 | Japan | Yes | Unclear | Yes | Yes | Yes | Yes | Yes |
| 168 | Shiraishi et al. [168] | 2021 | Japan | Yes | Yes | Yes | Yes | Yes | Unclear | Yes |
| 169 | Singh et al. [169] | 2015 | Republic of Korea | Yes | Yes | Yes | Yes | Yes | Yes | Yes |
| 170 | Skiadas et al. [170] | 2009 | Greece | Yes | Unclear | Yes | Yes | Yes | No | Yes |
| 171 | Stanek et al. [171] | 2007 | Czech Republic | Yes | Yes | Yes | Yes | Yes | Yes | No |
| 172 | Staple [172] | 1972 | USA | Yes | Yes | Yes | Yes | Yes | Yes | No |
| 173 | Stuplich et al. [173] | 2005 | Switzerland | Yes | Yes | Yes | Yes | Yes | Yes | Yes |
| 174 | Sugiura et al. [174] | 2004 | Japan | Yes | Yes | Yes | Yes | Yes | Yes | Yes |
| 175 | Sun et al. [175] | 2009 | Republic of Korea | Yes | Yes | Yes | Yes | Yes | Yes | No |
| 176 | Suzuki et al. [176] | 2021 | USA | Yes | Yes | Yes | Yes | Yes | Yes | Yes |
| 177 | Tamaki et al. [177] | 2017 | Japan | Yes | Yes | Yes | Yes | Yes | Yes | Yes |
| 178 | Tani et al. [178] | 2001 | Japan | Yes | Yes | Yes | Yes | Yes | Yes | Yes |
| 179 | Tateiwa et al. [179] | 2009 | Japan | Yes | Yes | Yes | Yes | Yes | Yes | Yes |
| 180 | Tatsumura et al. [180] | 2008 | Japan | Yes | Yes | Yes | Yes | Yes | Yes | No |
| 181 | Tebib et al. [181] | 1987 | France | Yes | Yes | Yes | Yes | Yes | Yes | Yes |
| 182 | Thienpoint and Vernaeve [182] | 2003 | Belgium | Yes | Yes | Yes | Yes | Yes | Yes | Yes |
| 183 | Tokita et al. [183] | 2008 | Japan | Yes | Yes | Yes | Yes | Yes | Yes | Yes |
| 184 | Toohey et al. [184] | 1990 | USA | Yes | Unclear | Yes | Yes | Yes | No | Yes |
| 185 | Torisu et al. [185] | 1978 | Japan | Yes | Yes | Yes | Yes | Yes | Yes | Yes |
| 186 | Tormenta et al. [186] | 2012 | Italy | Unclear | Unclear | Unclear | Yes | Yes | Unclear | Yes |
| 187 | Tsuji et al. [187] | 2016 | Japan | Yes | Yes | Yes | Yes | Yes | Yes | Yes |
| 188 | Underwood et al. [188] | 1988 | USA | Yes | No | Yes | Yes | Yes | Unclear | Yes |
| 189 | Van de Perre et al. [189] | 2005 | Belgium | Yes | Yes | Yes | Yes | No | No | Yes |
| 190 | Van Mourik et al. [190] | 1988 | The Netherlands | Yes | Yes | Yes | Yes | Yes | Yes | No |
| 191 | Van Riet et al. [191] | 1996 | The Netherlands | Yes | No | Yes | Yes | Yes | Yes | Yes |
| 192 | Vohra et Jones [192] | 2000 | UK | Yes | Yes | Yes | Yes | Yes | No | Yes |
| 193 | Wang et al. [193] | 2018 | China | Yes | Yes | Yes | Yes | Yes | Yes | Yes |
| 194 | Warren et al. [194] | 1975 | USA | Yes | Yes | Yes | Yes | Yes | Yes | No |
| 195 | Weber et al. [195] | 2008 | Switzerland | Yes | Yes | Yes | Yes | Yes | Yes | Yes |
| 196 | Weisser and Robinson [196] | 1951 | USA | Yes | Yes | Yes | Yes | Yes | Yes | Yes |
| 197 | White et al. [197] | 1988 | USA | Yes | Yes | Yes | Yes | Yes | Unclear | Yes |
| 198 | Wilkinson and Palmer [198] | 1991 | UK | Unclear | Yes | Yes | Yes | Yes | Unclear | No |
| 199 | Williams and Marks [199] | 1978 | UK | Yes | Yes | Yes | Yes | Yes | No | Yes |
| 200 | Wilson et al. [200] | 2000 | USA | Yes | Unclear | Unclear | Yes | Unclear | No | No |
| 201 | Winston and Henley [201] | 1954 | UK | Yes | Yes | Yes | Yes | Yes | Yes | Yes |
| 202 | Winter et al. [202] | 2021 | USA | Yes | Yes | Yes | Yes | Yes | Yes | No |
| 203 | Wu and Liu [203]1986 | 1986 | Taiwan | Yes | Yes | Yes | Yes | Yes | Yes | Yes |
| 204 | Wuenschel and Kunze [204] | 2011 | Germany | Yes | Yes | Yes | Yes | Yes | Yes | Yes |
| 205 | Wunderbaldinger et al. [205] | 2002 | Austria and Germany | Unclear | Unclear | Unclear | Yes | Yes | No | Yes |
| 206 | Yamamoto et al. [206] | 2021 | Japan | Yes | No | Yes | Yes | Yes | Unclear | Yes |
| 207 | Yamamoto et al. [207] | 2003 | Japan | Yes | Yes | Yes | Yes | Yes | Yes | Yes |
| 208 | Yang and Bronson[208] | 1993 | USA | Yes | Yes | Yes | Yes | Yes | Yes | Yes |
| 209 | Ye et al. [209] | 2019 | China | Yes | Yes | Yes | Yes | Yes | Yes | Yes |
| 210 | Yoon et al. [210] | 1995 | Republic of Korea | Yes | Yes | Yes | Yes | Yes | Yes | Yes |
| 211 | Yoon et al. [211] | 2000 | Republic of Korea | Yes | Yes | Yes | Yes | Yes | Yes | No |
| 212 | Yoshioka et al. [212] | 2008 | Japan | Yes | Yes | Yes | Yes | Yes | Yes | No |
| 213 | Zack et al. [213] | 2003 | USA | Yes | Yes | Yes | Yes | Yes | Yes | Yes |
| 214 | Hattrup et al. [214] | 1988 | USA | Yes | Yes | Yes | Yes | Yes | Yes | Yes |
| 215 | Shilt et al. [215] | 1997 | USA | Yes | Yes | Yes | Yes | Yes | Unclear | Unclear |
| 216 | Hisatome et al. [216] | 2003 | Japan | Yes | Yes | Yes | Yes | Yes | Yes | Yes |
| 217 | Sumanovac [217] | 1959 | Yugoslavia | Yes | Yes | Yes | Yes | Yes | Yes | Yes |

**REFERENCES**

1. Albaba M. Acute debilitating iliopsoas bursitis in a elder patient diagnosed by computed tomography. *Journal of the American Geriatrics Society.* 2008;56:S165-S165.

2. Algarni AD, Huk OL, Pelmus M. Metallosis-induced iliopsoas bursal cyst causing venous obstruction and lower-limb swelling after metal-on-metal THA. *Orthopedics.* 2012;35:e1811-1814.

3. Al-Khodairy AT, Gobelet C, Nançoz R, De Preux J. Iliopsoas bursitis and pseudogout of the knee mimicking L2-L3 radiculopathy: Case report and review of the literature. *European Spine Journal.* 1997;6:336-341.

4. Alter TD, Wichman DM, Knapik DM, Bessa FS, Nho SJ. Iliopsoas Bursitis Managed with Endoscopic Bursectomy and Lesser Trochanter Decompression: A Case Report. *Jbjs Case Connector.* 2021;11:11.

5. Angelini A, Zanotti G, Berizzi A, Staffa G, Piccinini E, Ruggieri P. Synovial cysts of the hip. *Acta Biomedica.* 2017;88:483-490.

6. Armstrong P, Saxton H. Ilio-psoas bursa. *The British journal of radiology.* 1972;45:493-495.

7. Atkinson MH. Rheumatoid synovial cyst of the hip: an unusual cause of leg swelling. *The Journal of rheumatology.* 1986;13:986-988.

8. Avasarala SK, Ahsan ST. Bilateral lower-extremity edema caused by iliopsoas bursal distention: After hip arthroplasty. *Texas Heart Institute Journal.* 2016;43:550-551.

9. Bakx PAGM, Wiggers RH. Iliopsoas bursa: A rare type of swelling in the groin. *European Journal of Surgery, Acta Chirurgica.* 1996;162:249-250.

10. Beardsmore D, Spark JI, MacAdam R, Macdonald D, Scott DJA. A Psoas ganglion causing obstruction of the iliofemoral arteries. *European Journal of Vascular and Endovascular Surgery.* 2000;19:554-555.

11. Bekou V, Galis D, Traber J. Unilateral leg swelling: Deep vein thrombosis? *Phlebology.* 2011;26:8-13.

12. Beksaç B, Tözün R, Baktiroglu S, Şener N, Gonzalez Della Valle A. Extravascular Compression of the Femoral Vein Due to Wear Debris-Induced Iliopsoas Bursitis. A Rare Cause of Leg Swelling After Total Hip Arthroplasty. *Journal of Arthroplasty.* 2007;22:453-456.

13. Bergenudd H, Bengner U, Telhag H, Hjelmqvist B. Ganglion of the hip - an unusual cause of soft tissue swelling of the groin. *Archives of Orthopaedic and Traumatic Surgery.* 1987;106:274-275.

14. Bhargava A, Singh S, Shrivastava RK. Giant synovial cysts of the hip joint: An important but rare differential diagnosis of an inguinal swelling. *HIP International.* 2004;14:51-54.

15. Bianchi S, Martinoli C, Keller A, Bianchi-Zamorani MP. Giant iliopsoas bursitis: Sonographic findings with magnetic resonance correlations. *Journal of Clinical Ultrasound.* 2002;30:437-441.

16. Bilora F, Sarolo L, Pomerri F, Prandoni P. Iliopsoas bursitis and femoral vein thrombosis complicating total hip arthroplasty in an elderly patient. *Aging Clinical and Experimental Research.* 2017;29:1067-1069.

17. Binek R, Levinsohn EM. Enlarged iliopsoas bursa. An unusual cause of thigh mass and hip pain. *Clinical Orthopaedics and Related Research.* 1987;224:158-163.

18. Bobrowski A, Cseh A, Pschibul A, Schorling D, Moske-Eick O, Raedecke J, Zieger B. Successful Surgical Removal of A Massive Iliopsoas Pseudotumor in a Boy with Mild Hemophilia A. *Klinische Padiatrie.* 2018;230:333-335.

19. Bolhuis HW, Van Der Werf TS, Tjabbes T, Ponsen RJG, Van De Loo RAF. Giant synovial cyst of the hip joint presenting with femoral vein compression. *Netherlands Journal of Surgery.* 1990;42:88-91.

20. Brunot S, Dubeau S, Laumonier H, Creusé A, Delmeule T, Reboul G, Das Neves D, Bouin H. Acute inguinal pain associated with iliopectineal bursitis in four professional soccer players. *Diagnostic and Interventional Imaging.* 2013;94:91-94.

21. Burnett RA, Westermann R, Bedard N, Liu S, Callaghan JJ. Ganglion Cyst as a Rare Complication of Hip Arthroscopy Resolved With THA: A Case Report. *The Iowa orthopaedic journal.* 2018;38:87-91.

22. Butler RA, Barrack RL. Total hip wear debris presenting as lower extemity swelling. *J. Bone Joint Surg.-Am. Vol.* 2004;86A:142-145.

23. Byrne PAC, Rees JIS, Williams BD. Iliopsoas bursitis - An unusual presentation of metastatic bone disease. *British Journal of Rheumatology.* 1996;35:285-288.

24. Bystrom S, Adalberth G, Milbrink J. Giant synovial cyst of the hip: An unusual presentation with compression of the femoral vessels. *Canadian Journal of Surgery.* 1995;38:368-370.

25. Cantini F, Niccoli L, Salvarani C, Padula A, Olivieri I, Bozza A. Hip bursitis in active polymyalgia rheumatica: Report of a case [1]. *Clinical and Experimental Rheumatology.* 1999;17:512-513.

26. Cassina PC, Hauser M, Kossmann T, Brunner U. Juxtaacetabular ganglion as a differential diagnosis in pulsating groin masses. *Vasa - Journal of Vascular Diseases.* 2000;29:75-76.

27. Chaiamnuay P, Davis P. An unusual case of inguinal swelling. *Arthritis and rheumatism.* 1984;27:239-240.

28. Chalmers J CN. Leg oedema due to a rheumatoid cyst in the pelvis. *J Bone Joint Surg Br* 1992;74:390-392.

29. Chen WL, Tsai JC. Hip Ganglion Cyst Causing Femoral Nerve Dysesthesia. *American journal of physical medicine & rehabilitation.* 2017;96:e227.

30. Cheung YM, Gupte CM, Beverly MJ. Iliopsoas bursitis following total hip replacement. *Archives of Orthopaedic and Trauma Surgery.* 2004;124:720-723.

31. Chilton CP, Darke SG. External iliac venous compression by a giant iliopsoas rheumatoid bursa. *British Journal of Surgery.* 1980;67:641.

32. Cho MR, Jun CM, Oh TB, Kwon JB, Choi WK. Pseudotumor mimicking iliacus muscle abscess following a total hip arthoplasty with metal on metal articulation: A case report. *Medicine.* 2019;98:e16322.

33. Y. C. CT-guided aspiration and steroid injection of symptomatic paralabral cysts of the hip. *Jpn J Radiol.* 2015;33:2229-2232.

34. Clarke MD, Edwards DP, Barker P. Ganglion of the hip joint--we present a logical approach to the exploration of a mass in the femoral triangle. *Journal of the Royal Army Medical Corps.* 1999;145:145-146.

35. Cohen JM, Hodges SC, Weinreb JC, Muschler G. Case report. MR imaging of iliopsoas bursitis and concurrent avascular necrosis of the femoral head. *Journal of Computer Assisted Tomography.* 1985;9:969-971.

36. Colasanti M, Sapienza P, Moroni E, Mosiello G, Postacchini F, di Marzo L. An Unusual Case of Synovial Cyst of the Hip Joint Presenting as Femoral Vein Compression and Severe Lower Limb Edema. *European Journal of Vascular and Endovascular Surgery.* 2006;32:468-470.

37. Conroy J, Caus S, Nelms NJ. A Case of Iliopsoas Bursitis With Compressive Femoral Nerve Palsy Treated With Iliopsoas Tendon Release. *Arthroplasty Today.* 2022;14:148-153.

38. Cook TD. Ganglion of the hip; case report. *Surgery.* 1952;32:129-131.

39. Corvino A, Venetucci P, Caruso M, Tarulli FR, Carpiniello M, Pane F, Sabatino V, Franzese R, Catalano O, Corvino F, Catelli A. Iliopsoas bursitis: The role of diagnostic imaging in detection, differential diagnosis and treatment. *Radiology Case Reports.* 2020;15:2149-2152.

40. Coulier B, Cloots V. Atypical retroperitoneal extension of iliopsoas bursitis. *Skeletal Radiology.* 2003;32:298-301.

41. Coventry MB, Polley HF, Weiner AD. Rheumatoid synovial cyst of the hip; report of three cases. *The Journal of bone and joint surgery. American volume.* 1959;41 A:721-730 passim.

42. Czuczman GJ, Mandell JC, Khurana B. Iliopsoas bursal extension of lipohemarthrosis: A novel imaging finding associated with hip fracture. *Skeletal Radiology.* 2017;46:253-257.

43. Dan J, Okanoue Y, Kitaoka K, Ikeuchi M. Prevalence of iliopsoas bursitis in patients with end-stage hip osteoarthritis. *Modern rheumatology.* 2021.

44. DeFrancesco CJ, Kamath AF. Abductor muscle necrosis due to iliopsoas bursal mass after total hip arthroplasty. *Journal of Clinical Orthopaedics and Trauma.* 2015;6:288-292.

45. DeFrang RD, Guyer WD, Porter JM, Duwelius PJ. Synovial cyst formation complicating total hip arthroplasty: A case report. *Clinical Orthopaedics and Related Research.* 1996;325:163-167.

46. Di Benedetto P, Magnanelli S, Buttironi MM, Beltrame A, Causero A. Groin pain caused by iliopsoas synovial cyst treated with endoscopic approach. A case report. *Acta Biomedica.* 2019;90:174-177.

47. Di Carlo M, Draghessi A, Carotti M, Salaffi F. An Unusual Association: Iliopsoas Bursitis Related to Calcium Pyrophosphate Crystal Arthritis. *Case Reports in Rheumatology Print.* 2015;2015:935835.

48. Di Sante L, Paoloni M, De Benedittis S, Tognolo L, Santilli V. Groin pain and iliopsoas bursitis: Always a cause-effect relationship? *Journal of Back and Musculoskeletal Rehabilitation.* 2014;27:103-106.

49. DiMaio FR, Santore RF. A large ganglion cyst in a patient with hip dysplasia. *Orthopedics.* 1997;20:650-652.

50. Emery D, Griffiths W. Ileopectineal bursitis: Orthopaedic cause for a lump in the groin. *Journal of the Royal Society of Medicine.* 1997;90:158-159.

51. Emura T, Yokomori K, Obana K, Tanaka Y. Ganglion of the groin in a child: An unusual cause of soft-tissue swelling of the groin. *Pediatric Surgery International.* 2005;21:227-229.

52. Endo M, Sato H, Murakami S, Kidani M, Noto T. A case of pseudothrombophlebitis due to inguinal synovial cyst. *American Surgeon.* 1990;56:533-534.

53. Enzler M, Drobny T, Franzeck U, Inderbitzi R, Leu A. Inguinal mass as a late complication of hip arthroplasty: Differential diagnosis and treatment from a vascular surgical perspective. *Vasa - Journal of Vascular Diseases.* 2000;29:288-291.

54. Farrington WJ, Lewis P, MacEachern AG. Giant synovial cyst causing femoral occlusion following a hip replacement. A case report and review of the literature. *HIP International.* 2002;12:394-396.

55. Finder J. Iliopectineal Bursitis. *Arch Surg.* 1938;36(3):519-530.

56. Flanagan FL, Sant S, Coughlan RJ, O'Connell D. Symptomatic enlarged iliopsoas bursae in the presence of a normal plain hip radiograph. *British Journal of Rheumatology.* 1995;34:365-369.

57. Fokter SK, Repse-Fokter A, Takac I. Case Report: Femoral Neuropathy Secondary to Total Hip Arthroplasty Wear Debris. *Clinical Orthopaedics and Related Research.* 2009;467:3032-3035.

58. Ford MJ, Martynoga AG, Nuki G. Iliopsoas bursitis in rheumatoid arthritis: An unusual cause of leg oedema. *British Medical Journal.* 1981;282:947-948.

59. Forster BB, Connell DG, Scudamore CH. Synovial cyst of the hip: An unusual cause of an inguinal mass. *Canadian Journal of Surgery.* 1989;32:133-134.

60. Fortin L, Belanger R. Bursitis of the iliopsoas: Four cases with pain as the only clinical indicator. *Journal of Rheumatology.* 1995;22:1971-1973.

61. Fukui K, Kaneuji A, Kawahara N. External iliac vein compression and lower-extremity swelling caused by an iliopectineal ganglion: A case report. *Journal of Medical Case Reports.* 2019;13.

62. Fukui S, Iwamoto N, Tsuji S, Umeda M, Nishino A, Nakashima Y, Suzuki T, Horai Y, Koga T, Kawashiri SY, Ichinose K, Hirai Y, Tamai M, Nakamura H, Origuchi T, Kawakami A. RS3PE syndrome with iliopsoas bursitis distinguished from an iliopsoas abscess using a CT-guided puncture. *Internal Medicine.* 2015;54:1653-1656.

63. Gale SS, Fine M, Dosick SM, Whalen RC. Deep vein obstruction and leg swelling caused by femoral ganglion. *Journal of Vascular Surgery.* 1990;12:594-595.

64. Gatch WD, Green, W.T. Cysts of the ilio-psoas bursa *Annals of surgery.* 1925.

65. Generini S, Matucci-Cerinic M. Iliopsoas bursitis in rheumatoid arthritis. *Clinical and Experimental Rheumatology.* 1993;11:549-551.

66. Ginesty E, Dromer C, Galy-Fourcade D, Benazet JF, Marc V, Zabraniecki L, Railhac JJ, Fournie B. Iliopsoas bursopathies. A review of twelve cases. *Revue du Rhumatisme (English Edition).* 1998;65:181-186.

67. Gomori A, Gombos J, Papp M. An unusual case of hip pain after total hip arthroplasty: A case report. *Joint Diseases and Related Surgery.* 2021;32:546-550.

68. Gong W, Ge F, Chen L. A giant ganglion cyst of hip joint causing lower limb edema. *Saudi Medical Journal.* 2010;31:569-571.

69. Goupille P, Anger C, Burdin P, Valat JP. Iliopsoas bursitis. *Journal of Rheumatology.* 1990;17:1566-1567.

70. Grindulis KA, McConkey B, Norcross K. Iliopsoas bursitis - a surgically correctable cause of lower limb oedema. *Practitioner.* 1982;226:1336-1337.

71. F.W. G. Cystic lesion of the groin due to metallosis a rare long-term complication of metal-on-metal total hip arthroplasty. *J Arthroplasty.* 2007;22:923-927.

72. Harris RW, Andros G, Dulawa LB, Oblath RW, Horowitz R. Iliofemoral venous obstruction without thrombosis. *Journal of Vascular Surgery.* 1987;6:594-599.

73. Harris JM, North Jr JH, Hamelink JK. The utility of ultrasonography in the evaluation of groin masses: A case report. *American Surgeon.* 1997;63:1002-1004.

74. Hauptfleisch J, Pandit H, Grammatopoulos G, Gill HS, Murray DW, Ostlere S. A MRI classification of periprosthetic soft tissue masses (pseudotumours) associated with metal-on-metal resurfacing hip arthroplasty. *Skeletal Radiology.* 2012;41:149-155.

75. Helfgott SM. Unusual features of iliopsoas bursitis. *Arthritis and rheumatism.* 1988;31:1331-1333.

76. Holton J, Palmer A, Kendrick B, Ramsden A, Taylor A, Glyn-Jones S. Iliopsoas cyst: Rare cause of hip pain and vascular compression in osteoarthritis. *BMJ (Online).* 2014;349.

77. Howie DW, Cain CMJ, Cornish BL. Pseudo-abscess of the psoas bursa in failed double-cup arthroplasty of the hip. *Journal of Bone and Joint Surgery - Series B.* 1991;73:29-32.

78. Hung CY, Chang KV, Özçakar L. Avascular Necrosis of the Femoral Head Masquerading as Iliopsoas Bursitis: Imaging With Ultrasound and Magnetic Resonance. *American journal of physical medicine & rehabilitation / Association of Academic Physiatrists.* 2016;95:e24-e25.

79. Huang KY, Yang RS, Hsieh CC. An iliopsoas ganglion mimicking femoral hernia. *Journal of Musculoskeletal Research.* 2014;17.

80. Huang HT, Tsai IC, Cheng SB, Chen CCC. Unilateral lower limb swelling caused by a synovial cyst of the hip joint. *Tzu Chi Medical Journal.* 2010;22:65-67.

81. Ikard RW. Synovial cysts of the groin. Case report. *Acta Chirurgica - European Journal of Surgery.* 1991;157:285-287.

82. Iversen JK, Nelleman H, Buus A, Stengaard-Pedersen K, Lucht U, Myhre Jensen O, Jurik AG. Synovial cysts of the hips in seronegative arthritis. *Skeletal Radiology.* 1996;25:396-399.

83. Iwata T, Nozawa S, Ohashi M, Sakai H, Shimizu K. Giant iliopectineal bursitis presenting as neuropathy and severe edema of the lower limb: Case illustration and review of the literature. *Clinical Rheumatology.* 2013;32:721-725.

84. Janus C, Hermann G. Enlargement of the iliopsoas bursa: Unusual cause of cystic mass on pelvic sonogram. *Journal of Clinical Ultrasound.* 1982;10:133-135.

85. Jeremy R. Acute psoas bursitis and unusual joint cysts in rheumatoid arthritis. *The Medical journal of Australia.* 1969;2:1106-1107.

86. Jerosch J, Sokkar S, El-Tayar A, Sallam A. Minimum 5-year follow-up of arthroscopic treatment of symptomatic iliopectineal cyst. *European Journal of Orthopaedic Surgery and Traumatology.* 2021;31:1369-1374.

87. Jones PBB, Economou G, Adams JE, Bernstein RM. Iliopsoas bursa presenting as deep vein thrombosis in rheumatoid arthritis. *British Journal of Rheumatology.* 1993;32:832-834.

88. Jung JH, Song GG, Kim JH. Sarcoidosis Presenting as Multifocal Bursitis. *Journal of the College of Physicians and Surgeons--Pakistan : JCPSP.* 2019;29:295-296.

89. KalacI A, Dogramaci Y, Sevinç T, Yanat A. Femoral nerve compression secondary to a ganglion cyst arising from a hip joint: A case report and review of the literature. *Journal of Medical Case Reports.* 2009;3.

90. Kanauchi T, Suganuma J, Mochizuki R, Uchikawa S. Arthroscopic Treatment of Femoral Nerve Paresthesia Caused by an Acetabular Paralabral Cyst. *Orthopedics.* 2014;37:E496-E499.

91. Kataoka M, Torisu T, Nakamura M, Uchida K. Iliopsoas bursa of the rheumatoid hip joint. A case report and review of the literature. *Clinical Rheumatology.* 1995;14:358-364.

92. Kawakita K, Shibanuma N, Tei K, Nishiyama T, Kuroda R, Kurosaka M. Leg Edema Due to a Mass in the Pelvis After a Large-Diameter Metal-On-Metal Total Hip Arthroplasty. *Journal of Arthroplasty.* 2013;28:197.e191-197.e194.

93. Kawasaki M, Inoue H, Sabanai K, Sawai T, Sato K. Synovial cyst of the hip in a patient with rheumatoid arthritis. *Modern Rheumatology.* 2013;23:587-592.

94. Keese M, Dahi F, Lindhoff-Last E. A Synovial Cyst Originating from the Hip Joint as a Rare Cause of Recurrent Femoral Vein Thrombosis: Case Report and Literature Review. *Annals of Vascular Surgery.* 2017;43:313.e313-313.e315.

95. Kenaan MK, Wynn-Jones CH, Cassar-Pullicino VN. Ilio-psoas bursitis presenting as deep vein thrombosis. *HIP International.* 1999;9:154-157.

96. Kerry R, King DG, Gibson MF. Iliopsoas bursitis: Physical diagnosis and management with ultrasonography and corticosteroid infiltration in a 33 year-old man. *Physiotherapy.* 2000;86:306-311.

97. Kim S, Lee HJ, Park JH, Kim T, Nam K. Tarlov Cysts Misdiagnosed as Adnexal Masses in Pelvic Sonography: A Literature Review. *Frontiers in Medicine.* 2020;7.

98. Kim JO, Cho HM. Rapid Destruction of the Hip Joint Accompanied by an Enlarged Iliopsoas Bursa in a Healthy Man. *Hip & Pelvis.* 2014;26:189-193.

99. Kim HK, Hwang D, Park S, Jeong WJ, Seo AN, Huh S. Cystic Disease of the Groin Presenting as Compression of a Femoral Vessel. *Vascular Specialist International.* 2016;32:124-128.

100. Kolmert L, Persson BM, Herrlin K, Ekelund L. Ileopectineal bursitis following total hip replacement. *Acta Orthopaedica Scandinavica.* 1984;55:63-65.

101. D. K. Large osteoarthritic cyst presenting as soft tissue tumour - a case repor. *Ann R Coll Surg Engl* 2007;89:4-6.

102. Kozlov DB, Sonin AH. Iliopsoas bursitis: Diagnosis by MRI. *Journal of Computer Assisted Tomography.* 1998;22:625-628.

103. Kuroyanagi G, Yamada K, Imaizumi T, Mizutani J, Wada I, Kozawa O, Tokuda H, Otsuka T. Leg lymphedema caused by iliopectineal bursitis associated with destruction of a rheumatoid hip joint: A case report. *Experimental and Therapeutic Medicine.* 2013;6:887-890.

104. Kurze C, Keel M, Siebenrock K, Attinger M. lliopsoas Muscle Necrosis Caused by Intrapelvic Extension of an Iliopectineal Bursitis Complicating Femoral Head Necrosis: Case Report and Literature Review. *Swiss Medical Weekly.* 2014;144:45S-45S.

105. Lavyne MH, Voorhies RM, Coll RH. Femoral neuropathy caused by an iliopsoas bursal cyst. Case report. *Journal of Neurosurgery.* 1982;56:584-586.

106. Lax Pérez R, Salinas Gilabert JE, Lajara Marco F, Lax Pérez A, Ferrero Manzanal F, García-Gálvez A, Izquierdo Plazas L. Femoral superficial vein thrombosis due to a large iliopsoas bursitis secondary to polyethylene wear debris in total hip arthroplasty. *Revista Espanola de Cirugia Ortopedica y Traumatologia.* 2012;56:54-58.

107. Leekam RN, Matzinger MA, Mustard RA, Grosman H. Enlarged iliopsoas bursa simulating neoplasm on sonographic examination. *Journal of Ultrasound in Medicine.* 1985;4:493-494.

108. Letourneau L, Dessureault M, Carette S. Rheumatoid iliopsoas bursitis presenting as unilateral femoral nerve palsy. *Journal of Rheumatology.* 1991;18:462-463.

109. Leung P, Kudrna JC. Growth of an intrapelvic pseudotumor associated with a metal-on-metal total hip arthroplasty after revision arthroplasty causing a femoral nerve neuropathy. *Arthroplasty Today.* 2016;2:105-109.

110. Levy RN, Hermann G, Haimov M. Rheumatoid synovial cyst of the hip. *Arthritis and Rheumatism.* 1982;25:1382-1384.

111. Li C, Liu H, Wang C, Han Q, Wang Z, Qin Y, Wang J, Yu T. A rare case report: Enlarged iliopsoas cystic solid mass associated with femoral head necrosis induced by heavy alcohol consumption. *Medicine (United States).* 2017;96.

112. Lim IGS, Berger M, Bertouch J. An unusual cause of pain in both hips. *Annals of the Rheumatic Diseases.* 2003;62:510-511.

113. Liman J, Von Gottberg P, Bähr M, Kermer P. Femoral nerve palsy caused by ileopectineal bursitis after total hip replacement: A case report. *Journal of Medical Case Reports.* 2011;5.

114. Lin YK, Tien YC, Lin SY. Hip ganglion cyst associated with developmental dysplasia of hip in a child - A case report. *Acta Orthopaedica Scandinavica.* 2002;73:109-110.

115. Lin YM, Ho TF, Lee TS. Iliopectineal bursitis complicating hemiarthroplasty: A case report. *Clinical Orthopaedics and Related Research.* 2001;392:366-371.

116. Loneragan R, Anderson J, Taylor J. Distended iliopsoas bursa: Case reports and anatomical dissection. *Australasian Radiology.* 1994;38:331-335.

117. Lupetin AR, Daffner RH. Rheumatoid iliopsoas bursitis: MR findings. *Journal of Computer Assisted Tomography.* 1990;14:1035-1036.

118. Mährlein R, Weiand G, Schmelzeisen H. Ganglion of the hip: report of five cases. *Journal of the Southern Orthopaedic Association.* 2001;10:1-5; discussion 5.

119. Matsumoto K, Hukuda S, Nishioka J, Fujita T. Iliopsoas bursal distension caused by acetabular loosening after total hip arthroplasty: A rare complication of total hip arthroplasty. *Clinical Orthopaedics and Related Research.* 1992;279:144-148.

120. Matsumoto H, Yamamoto E, Kamiya C, Miura E, Kitaoka T, Suzuki J, Deguchi J, Yamada H, Matsumoto R, Kuroda T, Sato O. Femoral vein compression resulting from a ganglion of the hip joint: a case report. *Avd.* 2012;5:233-236.

121. Matsumoto T, Juji T, Mori T. Enlarged psoas muscle and iliopsoas bursitis associated with a rapidly destructive hip in a patient with rheumatoid arthritis. *Modern Rheumatology.* 2006;16:52-54.

122. Maurer-Ertl W, Friesenbichler J, Liegl-Atzwanger B, Kuerzl G, Windhager R, Leithner A. Noninflammatory pseudotumor simulating venous thrombosis after metal-on-metal hip resurfacing. *Orthopedics.* 2011;34:e678-e681.

123. McGraw RW, Morton KS, Duncan CP. Massive intrapelvic synovial cyst as a complication of total hip replacement arthroplasty: A case report. *Canadian Journal of Surgery.* 1991;34:267-269.

124. McLaughlin GE. Sudden death in rheumatoid arthritis: Pulmonary embolism - A fatal complication of iliopsoas bursitis. *Journal of Clinical Rheumatology.* 2002;8:208-211.

125. Meaney JF, Cassar-Pullicino VN, Etherington R, Ritchie DA, McCall IW, Whitehouse GH. Ilio-psoas bursa enlargement. *Clinical Radiology.* 1992;45:161-168.

126. Melamed A, Bauer CA, Johnson JH. Iliopsoas bursal extension of arthritic disease of the hip. *Radiology.* 1967;89:54-58.

127. Monaghan N, Nwawka OK, Wyss JF. Giant iliopsoas bursa presenting as a large pulsatile groin mass. *PM and R.* 2014;6:857-859.

128. Morales-González JM, Riera-Rodríguez L, Novillo-Casal D. Persistent lower limb edema caused by synovial cysts in a patient with rheumatoid arthritis. *Revista de Ortopedia y Traumatologia.* 2005;49:443-446.

129. Mori S, Tamura T, Komatsubara S, Kawaguchi Y, Todo S, Inoo M, Kurata N, Norimatsu H. A case of femoral nerve palsy caused by iliopectineal bursitis associated with rheumatoid arthritis. *Modern Rheumatology.* 2004;14:274-278.

130. Morita M, Yamada H, Terahata S, Tamai S, Shinmei M. Pseudo-synovial cyst arising at the pubic bone region and forming a large femoral-inguinal mass. *Journal of Rheumatology.* 1997;24:396-399.

131. Murphy CL, Meaney JFM, Rana H, McCarthy EM, Howard D, Cunnane G. Giant iliopsoas bursitis: A complication of chronic arthritis. *Journal of Clinical Rheumatology.* 2010;16:83-85.

132. Nasra MH, Michel CR, Sudah S, Dijanic C, Torpey B. Anterior Hip Dislocation After Hip Arthroscopy Complicated by Iliopsoas Bursitis. *Cureus.* 2021;13:e17044.

133. Natsume K, Yamamoto K, Tanaka K, Hiraiwa T, Tanaka K. A Case of External Compression of Femoral Vein by the Enlarged Iliopsoas Bursa with Long Term Edema. *Avd.* 2015;8:100-102.

134. D.G. N. Management of a pelvic mass following a worn uncemented total hip arthroplasty. *J Arthroplasty.* 2011;27.

135. Nihal A, Drabu KJ. A lump in the groin: An unusual presentation of loose hip prosthesis. *Journal of the Royal College of Surgeons of Edinburgh.* 1998;43:59-60.

136. O'Connor DS. Early recognition of iliopectineal bursitis. *Surg. Gynecol. Obstet.* 1933;57:674-684.

137. O'Riordan CO, Ahmed W, Grace P, Burke T. Synovial cyst of the hip joint: an unusual cause of a pulsating groin mass. *Irish medical journal.* 2002;95:24-25.

138. Ornetti P, Turcu A, Vinit J. Clinical Images: Calcific iliopsoas bursitis in a patient with scleroderma mimicking avascular hip osteonecrosis. *Arthritis and Rheumatism.* 2010;62:1562.

139. Oshima S, Sumen Y, Yamasaki T, Ochi M. Arthroscopic Treatment for Femoral Nerve Palsy Associated with Ganglion Cyst of the Hip: A Case Report. *Journal of Orthopaedic Case Reports.* 2018;8:74-77.

140. Oshima J, Imai Y, Sasaki K, Sekido M. Giant Ganglion Cyst Arising from Iliac Wing, an Atypical Site. *Indian Journal of Plastic Surgery.* 2021;54:244-245.

141. Pachore JA, Shah VI, Upadhyay S, Shah K, Seth A, Kshatriya A. Compressive FemoralMononeuropathy Secondary to Acetabular Labral Tear Associated With Paralabral Ganglion Cyst of an Osteoarthritic Hip. *JBJS Case Connector.* 2019;9:E0344.

142. Pandit H. Pseudotumours associated with metal-onmetal hip resurfacings. *J Bone Joint Surg.* 2008;90-B:847-851.

143. Parfitt DJ, Wood SN, Chick CM, Lewis P, Rashid MH, Evans AR. Common Femoral Vein Thrombosis Caused By a Metal-On-Metal Hip Arthroplasty-Related Pseudotumor. *Journal of Arthroplasty.* 2012;27:1581e1589-1581e1511.

144. Park KS, Diwanji SR, Kim HK, Song EK, Yoon TR. Hemorrhagic Iliopsoas Bursitis Complicating Well-Functioning Ceramic-on-Ceramic Total Hip Arthroplasty. *Journal of Arthroplasty.* 2009;24:826.e821-826.e825.

145. Parziale JR, O'Donnell CJ, Sandman DN. Iliopsoas bursitis. *American journal of physical medicine & rehabilitation / Association of Academic Physiatrists.* 2009;88:690-691.

146. Patkar D, Shah J, Prasad S, Patankar T, Gokhale S, Krishnan A, Limdi J. Giant rheumatoid synovial cyst of the hip joint: diagnosed by MRI. *Journal of postgraduate medicine.* 1999;45:118-119.

147. Pellman E, Greenwald R. RHEUMATOID ILIOPSOAS BURSITIS - REPLY. *Journal of Rheumatology.* 1986;13:988-988.

148. Penkawa RR. Iliopsoas bursitis demonstrated by computed tomography. *American Journal of Roentgenology.* 1980;135:175-176.

149. Persson A, Eisler T, Bodén H, Krupic F, Sköldenberg O, Muren O. Revision for symptomatic pseudotumor after primary metal-on-polyethylene total hip arthroplasty with a standard femoral stem. *Journal of Bone and Joint Surgery - American Volume.* 2018;100:942-949.

150. Peters JC, Coleman BG, Turner ML. CT evaluation of enlarged iliopsoas bursa. *American Journal of Roentgenology.* 1980;135:392-394.

151. Pritchard RS, Shah HR, Nelson CL, FitsRandolph RL. MR and CT appearance of iliopsoas bursal distention secondary to diseased hips. *Journal of Computer Assisted Tomography.* 1990;14:797-800.

152. Ramage JS, Morton GB. Two cases of iliopsoas bursitis. *British Journal of Surgery.* 1934;21:705-708.

153. Ramsay AH, Donnelly PK. Non-invasive diagnosis of groin mass as hip ganglion using computerized tomography: A case report. *Asian Journal of Surgery.* 1999;22:409-410.

154. Raymond L, Christiansen S. Gas-containing iliopsoas bursitis in the setting of degenerative osteoarthritis. *BMJ Case Reports.* 2020;13.

155. Regis D. Recurrent femoral deep vein thrombosis rare complication of a pelvic mass induced by polyethylene wear debris following total hip arthroplasty. A case report. *Thromb Res.* 2008;121:593-595.

156. Ricci V, Özçakar L. Ultrasound Imaging for Anterior Hip Pain: Hypertrophic Bursitis between the Direct Tendon of the Rectus Femoris and the Iliocapsularis Muscle. *PM and R.* 2019;11:1031-1033.

157. Robinson KP, Carroll FA, Bull MJ, McClelland M, Stockley I. Transient femoral nerve palsy associated with a synovial cyst of the hip in a patient with spinal cord injury. *Journal of Bone and Joint Surgery - Series B.* 2007;89:107-108.

158. Rodriguez-Gomez M, Willisch A, Fernandez L, Lopez-Barros G, Abel V, Monton E. Bilateral giant iliopsoas bursitis presenting as refractory edema of lower limbs. *Journal of Rheumatology.* 2004;31:1452-1454.

159. Salmerón I, Cárdenas JL, Ramirez-Escobas MA, Bermejo C. Idiopathic iliopsoas bursitis. *European radiology.* 1999;9:175.

160. Samuelson C, Ward JR, Albo D. Rheumatoid synovial cyst of the hip. A case report. *Arthritis and rheumatism.* 1971;14:105-108.

161. Saraiva L, Eugénio G, Duarte C. Iliopectineal Bursitis in a Patient with Spondyloarthritis. *Journal of Clinical Rheumatology.* 2021;27:S620-S621.

162. Sartoris DJ, Danzig L, Gilula L. Synovial cysts of the hip joint and iliopsoas bursitis: A spectrum of imaging abnormalities. *Skeletal Radiology.* 1985;14:85-94.

163. Savarese RP, Kaplan SM, Calligaro KD, DeLaurentis DA. Iliopectineal bursitis: An unusual cause of iliofemoral vein compression. *Journal of Vascular Surgery.* 1991;13:725-727.

164. Schnarkowski P, Steinbach LS, Tirman PF, Peterfy CG, Genant HK. Magnetic resonance imaging of labral cysts of the hip. *Skeletal Radiology.* 1996;25:733-737.

165. Seo JS, Youm JW, Kim SM. Femoral Nerve Palsy due to Noninfectious Iliopsoas Bursitis and Hematoma after Total Hip Arthroplasty: A Case Report. *Hip & Pelvis.* 2018;30:125-128.

166. Seung-Bae H. Iliopsoas Bursitis with Compression of the Common Femoral Vein Resulting in Acute Lower Leg Edema. *J Korean Radiol Soc.* 2006;55:173-176.

167. Shimbo A, Akutsu Y, Yamazaki S, Shimizu M, Mori M. Giant Iliopsoas Bursitis in Systemic Juvenile Idiopathic Arthritis. *Arthritis and Rheumatology.* 2021;73:1328.

168. Shiraishi Y, Kanzawa Y, Ishimaru N, Kinami S. Iliopsoas bursitis related to calcium pyrophosphate deposition disease. *Internal Medicine.* 2021;60:2515-2516.

169. Singh V, Shon WY, Lakhotia D, Kim JH, Kim TW. A Rare Case of Femoral Neuropathy Associated with Ilio-Psoas Bursitis After 10 Years of Total Hip Arthroplasty. *The open orthopaedics journal.* 2015;9:270-273.

170. Skiadas V, Koutoulidis V, Plotas A. An atypical case of noninfected iliopsoas bursitis - MRI findings. *Journal of Radiology Case Reports.* 2009;3:15-18.

171. Stanek F, Ouhrabkova R, Hejdova H, Zubkovsky O, Ott Z, Kvasnicka J, Janousek M. Intermittent claudication caused by a hip joint ganglion. *Vasa - Journal of Vascular Diseases.* 2007;36:217-219.

172. Staple TW. Arthrographic demonstration of lliopsoas bursa extension of the hip joint. *Radiology.* 1972;102:515-516.

173. Stuplich M, Hottinger AF, Stoupis C, Sturzenegger M. Combined femoral and obturator neuropathy caused by synovial cyst of the hip. *Muscle and Nerve.* 2005;32:552-554.

174. Sugiura M, Komiyama T, Akagi D, Miyata T, Shigematsu H. Compression of the iliac vein by a synovial cyst. *Annals of Vascular Surgery.* 2004;18:369-371.

175. Sun WY, Jang LC, Park JW, Choi JW. Unilateral Leg Swelling Caused by a Ganglion Cyst on the Hip Joint. *J. Korean Surg. Soc.* 2009;76:333-335.

176. Suzuki Y, Poli de Figueiredo SM, Keyhani A, Tanaka A, Neely E, Keyhani K. Dual culprit for a swollen leg: Femoral vein compression caused by lymphadenopathy and a synovial cyst. *Journal of Vascular Surgery Cases, Innovations and Techniques.* 2021;7:734-736.

177. Tamaki Y, Goto T, Tsutsui T, Takasago T, Wada K, Sairyo K. Compression of the Femoral Vessels by a Pseudotumor after Metal-on-Metal Total Hip Arthroplasty. *Case Reports in Orthopedics.* 2017;2017:2594902.

178. Tani Y, Nishimura I, Mimura T, Ushiyama T, Inoue K, Murakami M. Enlargement of iliopsoas bursa in a patient with polymyalgia rheumatica [3]. *Journal of Rheumatology.* 2001;28:1198-1199.

179. Tateiwa T, Shinmura K, Ko M, Mibe J, Yamamoto K. Iliopectineal bursitis associated with rapid destruction of a rheumatoid hip joint. *Journal of Orthopaedic Science.* 2009;14:455-458.

180. Tatsumura M, Mishima H, Shiina I, Hara Y, Nishiura Y, Ishii T, Ochiai N, Ishii W, Sumida T. Femoral nerve palsy caused by a huge iliopectineal synovitis extending to the iliac fossa in a rheumatoid arthritis case. *Modern Rheumatology.* 2008;18:81-85.

181. Tebib JG, Dumontet C, Carret JP, Colson F, Bouvier M. Synovial cyst of the hip causing iliac vein and femoral nerve compression. *Clinical and experimental rheumatology.* 1987;5:92-93.

182. E. T. Reactive pelvic cyst following total hip arthroplasty. A case report. *Acta Orthop Belg.* 2003;69:292-294.

183. Tokita A, Ikari K, Tsukahara S, Toki H, Miyawaki M, Mochizuki T, Kawamura K, Tomatsu T, Momohara S. Iliopsoas bursitis-associated femoral neuropathy exacerbated after internal fixation of an intertrochanteric hip fracture in rheumatoid arthritis: A case report. *Modern Rheumatology.* 2008;18:394-398.

184. Toohey AK, LaSalle TL, Martinez S, Polisson RP. Iliopsoas bursitis: Clinical features, radiographic findings, and disease associations. *Seminars in Arthritis and Rheumatism.* 1990;20:41-47.

185. Torisu T, Chosa H, Kitano M. Rheumatoid synovial cyst of the hip joint. A case report. *Clinical Orthopaedics and Related Research.* 1978;137:191-194.

186. Tormenta S, Sconfienza LM, Iannessi F, Bizzi E, Massafra U, Orlandi D, Migliore A. Prevalence Study of Iliopsoas Bursitis in a Cohort of 860 Patients Affected by Symptomatic Hip Osteoarthritis. *Ultrasound in Medicine and Biology.* 2012;38:1352-1356.

187. Tsuji Y, Kitano I, Matsumoto S, Sawada K. Unilateral Leg Swelling Caused by Common Femoral Vein Compression by a Hip Ganglion Cyst in the Groin. *EJVES Short Reports.* 2016;33:20-23.

188. Underwood PL, McLeod RA, Ginsburg WW. The varied clinical manifestations of iliopsoas bursitis. *Journal of Rheumatology.* 1988;15:1683-1685.

189. Van De Perre S, Vanwambeke K, Vanhoenacker FM, De Schepper AM. Posttraumatic iliopsoas bursitis. *Journal Belge de Radiologie.* 2005;88:154-155.

190. Van Mourik JBA, Josaputra HA, Axler A. Giant synovial cyst causing deep venous thrombosis: Brief report. *Journal of Bone and Joint Surgery - Series B.* 1988;70:841.

191. Van Riet YEA, Van Vroonhoven TJMV, Van Der Werken C, Berkhoudt AN. Bursae communicating with the hip joint. A report on 2 cases. *Acta Orthopaedica Belgica.* 1996;62:120-122.

192. Vohra HA, Jones B. Femoral vein obstruction with an arthritic hip. *Journal of the Royal Society of Medicine.* 2000;93:594-595.

193. Wang J, Shao J, Qiu C, Chen Y, Liu B. Synovial cysts of the hip joint: a single-center experience. *BMC surgery.* 2018;18:113.

194. Warren R, Kaye JJ, Salvati EA. Arthrographic demonstration of an enlarged iliopsoas bursa complicating osteoarthritis of the hip. A case report. *Journal of Bone and Joint Surgery - Series A.* 1975;57:413-415.

195. Weber M, Prim J, Lüthy R. Inguinal pain with limping: Iliopectineal bursitis as first sign of polymyalgia rheumatica. *Joint Bone Spine.* 2008;75:332-333.

196. Weisser JR, Robinson DW. Pigmented villonodular synovitis of iliopectineal bursa; a case report. *The Journal of bone and joint surgery. American volume.* 1951;33 A:988-992.

197. White TK, Incavo SJ, Moreland MS. Giant synovial cyst of the hip joint. *Orthopaedic Review.* 1988;17:609-612.

198. Wilkinson L, Palmer R. Bilateral iliopsoas bursitis in rheumatoid arthritis. *British Journal of Rheumatology.* 1991;30:68-69.

199. Williams RA, Marks LJ. Synovial cyst causing an inguinal mass. *British Medical Journal.* 1978;2:91-92.

200. Wilson S, Chandler R, Neitzschman HR. Radiology case of the month. A groin mass. Adductor muscle pseudotumor. *The Journal of the Louisiana State Medical Society : official organ of the Louisiana State Medical Society.* 2000;152:475-476.

201. Winston P, Henley FA. Supernumerary pectineus bursa causing femoral vein compression. *British medical journal.* 1954;1:629.

202. Winter LD, Helmig KC, Goodwyn PJ, Gehlert RJ. Compression of the Rectum, Bladder, and External Iliac Vein Due to Hip Arthroplasty-Related Pseudotumor. *Cureus.* 2021;13:e20671.

203. Wu CC, Liu TJ. An unusual ganglion communicating with hip joint resembling a femoral hernia. Case report. *Acta Chirurgica Scandinavica.* 1986;152:705-706.

204. Wuenschel M, Kunze B. Iliopsoas cyst causing persistent pain after total hip arthroplasty. *Orthopedics.* 2011;34.

205. Wunderbaldinger P, Bremer C, Schellenberger E, Cejna M, Turetschek K, Kainberger F. Imaging features of iliopsoas bursitis. *European Radiology.* 2002;12:409-415.

206. Yamamoto S, Mukai T, Fujita S, Morita Y. Iliopectineal bursitis. *Rheumatology (Oxford, England).* 2021.

207. Yamamoto T, Marui T, Akisue T, Yoshiya S, Hitora T, Kurosaka M. Dumbbell-shaped iliopsoas bursitis penetrating the pelvic wall: A rare complication of hip arthrodesis. A case report. *Journal of Bone and Joint Surgery - Series A.* 2003;85:343-345.

208. Yang SS, Bronson MJ. Cystic enlargement of the iliopsoas bursa causing venous obstruction as a complication of total hip arthroplasty: A case report. *Journal of Arthroplasty.* 1993;8:657-661.

209. Ye Y, Zhang C, Zhang D, Chen N, Song B, Wu S, Guo X. Diagnosis and surgical treatment of patients with femoral vein compression from hip joint synovial cyst. *Journal of Vascular Surgery: Venous and Lymphatic Disorders.* 2019;7:82-89.

210. Yoon TR, Rowe SM, Chung JY, Moon ES, Song EK. Enlarged iliopsoas bursa concurrent with idiopathic avascular necrosis of the femoral head: A case report and review of the literature. *Journal of Orthopaedic Surgery.* 1995;3:71-75.

211. Yoon TR, Song EK, Chung JY, Park CH. Femoral neuropathy caused by enlarged iliopsoas bursa associated with osteonecrosis of femoral head - A case report. *Acta Orthopaedica Scandinavica.* 2000;71:322-324.

212. Yoshioka T, Tachihara A, Koyama T, Iwakawa K, Sakane M, Nakamura H. Rapidly destruction of the hip joint associated with enlarged iliopsoas bursa in a patient with refractory rheumatoid arthritis. *Journal of Nippon Medical School.* 2008;75:233-238.

213. Zack JR, Greben C, Simon DW, Naidich JB. Iliac artery thrombosis secondary to a giant synovial cyst. *Orthopedics.* 2003;26:1153-1154.

214. Hattrup SJ. Pelvic mass causing vesical compression after total hip arthroplasty. Case report. *Clin Orthop Relat Res* 1988:184-189.

215. J.S. S. Pelvic mass secondary to polyethylene and titanium alloy wear debris resulting in recurrent deep vein thrombosis. *J Arthroplasty.* 1997;12:946-949.

216. Hisatome T. Hidden intrapelvic granulomatous lesions associated with total hip arthroplasty: a report of two cases. *J Bone Joint Surg Am.* 2003;85:708-710.

217. Sumanovac Z. Traumatic cyst in the hip joint; a case report. *J Bone Joint Surg Am.* 1959;41:175-178.
